# The Melanocortin System in Atlantic Salmon (*Salmo salar* L.) and Its Role in Appetite Control

**DOI:** 10.3389/fnana.2020.00048

**Published:** 2020-08-21

**Authors:** Tharmini Kalananthan, Floriana Lai, Ana S. Gomes, Koji Murashita, Sigurd Handeland, Ivar Rønnestad

**Affiliations:** ^1^Department of Biological Sciences, University of Bergen, Bergen, Norway; ^2^Research Center for Aquaculture Systems, National Research Institute of Aquaculture, Japan Fisheries Research and Education Agency, Tamaki, Japan; ^3^Norwegian Research Center, NORCE Environment, Bergen, Norway

**Keywords:** melanocortin system, Atlantic salmon, *melanocortin-4 receptor* (*mc4r*), *proopiomelanocortin* (*pomc*), *agouti-related protein* (*agrp*), food intake, brain, appetite control centers

## Abstract

The melanocortin system is a key neuroendocrine network involved in the control of food intake and energy homeostasis in vertebrates. Within the hypothalamus, the system comprises two main distinct neuronal cell populations that express the neuropeptides proopiomelanocortin (POMC; anorexigenic) or agouti-related protein (AGRP; orexigenic). Both bind to the melanocortin-4 receptor (MC4R) in higher order neurons that control both food intake and energy expenditure. This system is relatively well-conserved among vertebrates. However, in Atlantic salmon (*Salmo salar* L.), the salmonid-specific fourth round whole-genome duplication led to the presence of several paralog genes which might result in divergent functions of the duplicated genes. In the current study, we report the first comprehensive comparative identification and characterization of Mc4r and extend the knowledge of Pomc and Agrp in appetite control in Atlantic salmon. *In silico* analysis revealed multiple paralogs for *mc4r* (*a1*, *a2*, *b1*, and *b2*) in the Atlantic salmon genome and confirmed the paralogs previously described for *pomc* (*a1*, *a2*, and *b*) and *agrp* (*1* and *2*). All Mc4r paralogs are relatively well-conserved with the human homolog, sharing at least 63% amino acid sequence identity. We analyzed the mRNA expression of *mc4r*, *pomc*, and *agrp* genes in eight brain regions of Atlantic salmon post-smolt under two feeding states: normally fed and fasted for 4 days. The *mc4ra2* and *b1* mRNAs were predominantly and equally abundant in the hypothalamus and telencephalon, the *mc4rb2* in the hypothalamus, and *a1* in the telencephalon. All *pomc* genes were highly expressed in the pituitary, followed by the hypothalamus and saccus vasculosus. The *agrp* genes showed a completely different expression pattern from each other, with prevalent expression of the *agrp1* in the hypothalamus and *agrp2* in the telencephalon. Fasting did not induce any significant changes in the mRNA level of *mc4r*, *agrp*, or *pomc* paralogs in the hypothalamus or in other highly expressed regions between fed and fasted states. The identification and wide distribution of multiple paralogs of *mc4r*, *pomc*, and *agrp* in Atlantic salmon brain provide new insights and give rise to new questions of the melanocortin system in the appetite regulation in Atlantic salmon.

## Introduction

In vertebrates, food intake is controlled by the synergic actions of central and peripheral signaling pathways which provide information on ingestion and presence of food in the digestive tract and on the nutritional status (Volkoff, 2016; Rønnestad et al., 2017). In mammals, the melanocortin system is a key neuroendocrine network playing a pivotal role in regulating appetite and energy homeostasis. This system is mainly located within the hypothalamus where neurons expressing the melanocortin-4 receptor (MC4R) mediates either anorexigenic or orexigenic signals, thereby controlling the energy homeostasis of the animal (Nuzzaci et al., 2015). The MC4R neurons receive dual innervation from neurons expressing MCR agonists as melanocortin-stimulating hormones (α-, β-, γ-MSHs) and adrenocorticotropic hormone (ACTH), which derive from the posttranslational cleavage of proopiomelanocortin (POMC) peptide and from neurons expressing the antagonist agouti-related peptide (AGRP) (Ghamari-Langroudi et al., 2011; Kim et al., 2014; Nuzzaci et al., 2015). Furthermore, both POMC and AGRP neurons integrate peripheral endocrine signals and information on nutrient levels received through blood circulation or vagal afferent projections. In mammals, leptin was shown to play an anorexigenic role by increasing the excitability of POMC neurons and decreasing AGRP neuron action (Cowley et al., 2001; Baver et al., 2014); on the contrary, ghrelin plays an orexigenic (appetite stimulator) role by directly stimulating AGRP neurons and inhibiting POMC neurons (Riediger et al., 2003). This complex network has been well-described in mammals; however, in other vertebrates, such as teleost, little knowledge still exists.

Many of the neuropeptides and endocrine signals involved in appetite control in mammals have also been identified in teleost, although only a few have been functionally described.

The involvement of the Mc4r receptor in teleost energy balance was demonstrated in salmonids, and modulation of the Mc4r activity with the receptor antagonist (HS024 or SHU9119) or the agonist (MTII) increased or decreased, respectively, food intake in rainbow trout (*Oncorhynchus mykiss*) (Schjolden et al., 2009). In common carp (*Cyprinus carpio*), brain *mc4r* expression declined with fasting, while it surged with refeeding (Wan et al., 2012). Similarly, a winter fasting state also induced a lower expression of *mc4r* in the hypothalamus in Arctic charr (*Salvelinus alpinus*) (Striberny et al., 2015). In an extended study in spotted sea bass (*Lateolabrax maculatus*), incubation of isolated brain cells with α-MSH showed changes at *npy* and *agrp* levels and a downregulation of *mc4r* transcript levels during both short- and long-term fasting (Zhang et al., 2019). Both *in vitro* and *in vivo* experiments demonstrated that a naturally mutated Mc4r in Mexican cave fish (*Astyanax mexicanus*) is responsible for elevated appetite, growth, and starvation resistance as an adaptation to an environment with poor nutrient conditions (Aspiras et al., 2015). In contrast, food deprivation in barfin flounder (*Verasper moseri*) did not induce any changes of *mc4r* transcripts in the brain (Kobayashi et al., 2008).

Several studies have explored the involvement of Pomc in appetite control in teleost, and the results have suggested that its role may be species-specific. Intracerebroventricular administration (ICV) of the Mc4r agonist MTII downregulated *pomc* mRNA levels (Kojima et al., 2010), whereas intraperitoneal injection of cholecystokinin octapeptide (Kang et al., 2010) and leptin (Yan et al., 2016) upregulated *pomc* expression in the diencephalon, favoring enhanced anorexigenic action of Pomc. Starvation of zebrafish (*Danio rerio*) larvae resulted in a decrease in *pomc* expression levels, and refeeding after 2 days of fasting recovered *pomc* to the level of the control group (Liu et al., 2016). Under hyperglycemic conditions, rainbow trout resulted in increased hypothalamic *pomc* mRNA levels (Conde-Sieira et al., 2010; Otero-Rodiño et al., 2015), while 28 days of fasting downregulated *pomc* expression in the same species (Leder and Silverstein, 2006).

In Atlantic salmon (*Salmo salar*), brain *pomc* levels declined from 3 to 6 h of post-feeding of a single meal (Valen et al., 2011). In fully fed growth hormone (GH) transgenic coho salmon (*Oncorhynchus kisutch*), hypothalamic *pomc* mRNA decreased 4 h post-feeding, while there was no difference in the non-transgenic group (Kim et al., 2015). Similar results were also reported in GH transgenic zebrafish fasted for 2 days (Dalmolin et al., 2015). No changes in the *pomc* mRNA expression were observed in zebrafish (Opazo et al., 2019), barfin flounder (Takahashi et al., 2005), and goldfish (*Carassius auratus*) (Cerdá-Reverter et al., 2003b) under fasting regimes. In Atlantic halibut (*Hippoglossus hippoglossus*) larvae, the response of *pomc* to food deprivation and refeeding did not show a consistent expression pattern to explain their contribution to appetite control (Gomes et al., 2015).

The AGRP-mediated action on food intake seen in mammals appears to be conserved in teleost species. Ablation of Agrp1-expressing neurons and knockout of the *agrp1* gene showed that Agrp stimulates food consumption in zebrafish larvae (Shainer et al., 2019) or induces obesity in transgenic zebrafish overexpressing *agrp* (Song and Cone, 2007). An upregulation of *agrp* transcript was described in the larvae of the same species under fasting conditions (Song et al., 2003). GH transgenic common carp (Zhong et al., 2013) and coho salmon (Kim et al., 2015) showed increased hypothalamic *agrp1* mRNA and elevated food intake compared to the wild type. Moreover, fasting upregulated the hypothalamic *agrp* mRNA in goldfish (Cerdá-Reverter and Peter, 2003), coho salmon (Kim et al., 2015), sea bass (Agulleiro et al., 2014), mouth brooding African cichlid (*Astatotilapia burtoni*) (Porter et al., 2017), rainbow trout (Comesaña et al., 2017), seabream (*Sparus aurata*) larvae (Koch et al., 2019), Atlantic salmon (Kalananthan et al., 2020), and Ya-fish embryo (*Schizothorax prenanti*) (Wei et al., 2013). In contrast, an opposite action was described in the brain of common carp (Wan et al., 2012) and Atlantic salmon (Murashita et al., 2009; Valen et al., 2011).

The extensively described variations in the role of Mc4r receptor and neuropeptides Pomc and Agrp in teleost compared to mammals may be due to major physiological and environmental adaptation. Moreover, salmonids went through a salmonid-specific fourth round whole-genome duplication (Ss 4R WGD) around 80 million years ago (mya), leading to large genomic rearrangements and the presence of several paralog genes, which may result in divergent functions for the different paralogs (Takahashi and Kawauchi, 2006; Warren et al., 2014; Lien et al., 2016).

Atlantic salmon is an economically important species of aquaculture industry in Norway. Periods of 2–4 days’ fasting is a common practice during transport, handling, vaccination, and harvest of salmon to ensure a proper evacuation of the gut (Waagbø et al., 2017). Studying the impact of fasting on fish biology is essential to optimize the Atlantic salmon aquaculture practices with regard to the period of recovery, fish welfare, and feed utilization. In this study, we investigated the spatial gene expression of *mc4r, pomc*, and *agrp* genes and their paralogs in the brain of Atlantic salmon post smolts at fed and fasting (4 days) states. Our study provides a foundation for new insights on the role of *mc4r*, *pomc*, and *agrp* genes in appetite, feed intake, and fasting in Atlantic salmon.

## Materials and Methods

### Ethics Statement

The animal experiments were carried out in accordance with Norwegian Animal Research Authority regulations and approved by the local representative of Animal Welfare at the Department of Biological Sciences, University of Bergen, Norway.

### Experimental Design

In this study, Atlantic salmon post smolt of ca. 250 g were obtained from Engesund fish farm (Fitjar, Norway) and randomly distributed into two 2,000-L tanks (48 fish per tank) at the Industrial Lab (ILAB) in Bergen (Norway). Fish were reared in tanks supplied with flow through seawater (27 ppt; 16 L/min) at 10°C and oxygen saturation above 80%. Constant light (LD 24:0) was provided in accordance to common practice in commercial aquaculture to promote optimal growth and to inhibit unwanted sexual maturation (Hansen et al., 1992; Endal et al., 2000; Nordgarden et al., 2003; Fjelldal et al., 2012). Fish were fed continuously with commercial dry feed pellets (Biomar intro 75 HH 50 mg Q) using an automatic feeder. Oxygen saturation, temperature, and salinity were measured daily, and the fish were acclimatized for 3 weeks. After the acclimation period, the two tanks were randomly labeled into two experimental groups, fed, and fasted. Thereafter, 21 fish per tank (263 ± 13.06 g and 275.7 ± 15.68 g) were sampled as a baseline control. Next, one tank was kept under continuous feeding (fed group) with the same commercial dry pellet, whereas the other tank was fasted for 4 days (fasted group). After the 4 days, 27 fish were sampled from the fed group (280 ± 12.69 g) and 26 from the fasted group (246 ± 12.88 g). One fish was excluded from this group due to previous mild winter sore mark. Fish from the fed and the fasted group were collected and euthanized using an overdose of 200 mg/L of MS222 (Tricaine methanesulfonate, Scan-Vacc, Hvam, Norway) before and after the 4 days fed/fasted, respectively. Length and weight were recorded. The whole brains were rapidly collected and transferred into RNAlater solution (Invitrogen, Carlsbad, CA, United States), kept at 4°C overnight, and then stored in −80°C until further analysis.

### Condition Factor (*K*) Calculation

Condition factor (*K*) was used to analyze the fitness of the fish before (fed *n* = 21; fasted *n* = 21) and after (fed *n* = 27; fasted *n* = 26) the feeding experiment by using weight and length of the fish in the following equation:

$$K=100\,\frac{W}{L^{3}}$$

where *W* is the weight (g) and *L* is the length of the fish (cm) (Froese, 2006).

### Structural Analysis and Phylogenetic Comparison of Mc4r and Pomc in the Salmonidae Family

Mc4r and Pomc peptide sequences of 17 species representatives of ray-finned fishes (Actinopterygiian) were retrieved from NCBI GenBank^1^ and Ensembl^2^ : the Lepisosteidae spotted gar (*Lepisosteus oculatus*) as a species before the teleost specific WGD (Ts WGD) (around 320 mya) (Lien et al., 2016), the Osteoglossidae Asian arowana (*Scleropages formosus*) as one of the oldest teleost groups; three Cyprinidae, including goldfish and common carp as species that went through a very recent 4R WGD and zebrafish which did not; one Characidae, cave fish; seven Salmonidae species, including Atlantic salmon, rainbow trout, chinook salmon (*Oncorhynchus tshawytscha*), sockeye salmon (*Oncorhynchus nerka*), coho salmon, arctic char, and brown trout (*Salmo trutta*); the Esocidae northern pike (*Esox lucius*) as a sister group of salmonids that diverged before the Ss 4R WGD; and three Neoteleostei Atlantic cod (*Gadus morhua*), medaka (*Oryzias latipes*), and stickleback (*Gasterosteus aculeatus*) (Supplementary Figures 2 and 3). The human amino acid (AA) sequences were also included in the analysis. Multiple alignments were generated using MUSCLE from MEGAX (Hall, 2013) and edited using GeneDoc 2.7 software (Nicholas et al., 1997).

Mc4r transmembrane domains/helices (TMHI-TMHVII), extracellular loops (ECL1-ECL3), and intracellular loops (ICL1-ICL3) were retrieved from UniProt^3^ database. Potential cleavage sites of Pomc precursor were acquired using UniProt and ProP 1.0^4^ by using the full-length AA sequences.

The phylogenetic trees were predicted using the maximum likelihood (ML) method and based on the predicted full-length AA sequence of Mc4r and Pomc. The substitution model used in the phylogenetic analysis was determined by using the best-fit substitution model suggested by MEGAX. A Jones Taylor Thornton (JTT) and gamma distributed (G) matrix-based model was used to produce the phylogenetic tree for Mc4r, while a JTT and G with invariant sites (I) matrix-based model was used for Pomc (Hall, 2013). Tertiary protein structures of Atlantic salmon Mc4r were predicted using the IntFOLD5 (McGuffin et al., 2019) and human MC4R structure with AGRP (PDB entry 2IQV) was retrieved from UniProt. The images were edited, and disulfide bonds were predicted by PyMOL Molecular Graphics System v 2.3.^5^ Searches for Agrp in Atlantic salmon genomic database did not identify any novel paralogs in addition to the ones previously published by Murashita et al. (2009).

### Brain Dissection

The Atlantic salmon brain of fed (*n* = 6) and fasted group (*n* = 6) was randomly selected and dissected into eight regions for appetite gene expression analysis: olfactory bulb, telencephalon, midbrain, cerebellum, hypothalamus, saccus vasculosus, pituitary, and medulla oblongata/brain stem. To ensure high RNA yield and quality, the brain was placed on an ice block during dissection under a zoom stereomicroscope (Olympus SZ51) and cleaned from blood vessels. The pineal gland, olfactory bulb, telencephalon, brain stem, cerebellum, saccus vasculosus, hypothalamus, and midbrain were separated in this order (Figure 4A).

### RNA Extraction and cDNA Synthesis

Total RNA was extracted from each section of the brains by using TRI Reagent (Sigma-Aldrich, MO, United States) following the manufacturer’s protocol. A NanoDrop ND-1000 spectrophotometer (Thermo Fisher Scientific, MA, United States) and a 2100 Bioanalyzer with RNA 6000 Nano Kit (Agilent Technologies, CA, United States) were used to assess the quantity and the quality of the extracted total RNA, respectively. To avoid any remnants of genomic DNA, 5 or 10 μg of total RNA was treated with TURBO DNase-free Kit (Ambion Applied Biosystems, CA, United States) with 1 μl of DNase (2 Units/μl) in 10 or 30 μl reaction volume. The amount of total RNA and the reaction volume for DNase treatment was adjusted depending on the amount of total RNA availability per region. First-strand cDNA was synthesized from 2 μg of the total RNA sample using SuperScript III Reverse Transcriptase (Invitrogen, CA, United States) and Oligo(dT)_20_ (50 μM) primers in a total reaction volume of 20 μl.

### Quantitative RT-PCR Setup and Primer Design

The salmon mRNA of *mc4r* (*a1*, *a2*, *b1*, and *b2*), *pomc* (*a1*, *a2*, and *b*), and *agrp* (*1* and *2*) was quantified by real-time quantitative RT-PCR (qPCR). The qPCR primers were designed from Atlantic salmon gene sequences retrieved from GenBank database (Table 1 for accession numbers information). For each gene paralog, primer pairs were designed using Primer3^6^ and/or NCBI primer designing tool and synthesized by Sigma-Aldrich (St. Louis, MO, United States). The specific primers were designed spanning exon–exon junctions when possible. All primers were analyzed for quantitation cycle (Cq), primers efficiency (E), and melting peaks. All qPCR products were analyzed in a 2% agarose gel, purified using QIAquick Gel Extraction Kit (Qiagen, Hilden, Germany), and cloned into a pCR4-TOPO vector (Thermo fisher, Scientific, Waltham, MA, United States). Sequencing was performed at the University of Bergen Sequencing Facility (Bergen, Norway), and their identity was confirmed using blastn analysis against the Atlantic salmon genome database.

**TABLE 1:** Primers sequences used for quantitative RT-PCR (qPCR) mRNA expression analysis in Atlantic salmon.

Gene	Gene Bank ID	Primer Sequence (5′ → 3′)	Amplicon size (bp)	*R*^2^	Efficiency %
*pomca1*	NM_001198575.1	ATACTTTTGAAACAGCGTGACGA	108	0.9985	103
		CAACGAGGATTCTCCCAGCA			
*pomca2*	NM_001198576.1	TTTGGCGACAGGCGAAGATG	91	0.999	98
	AB462420.1	TCCCAGCACTGACCTTTCAC			
*pomcb*	NM_001128604.1	CAGAGGACAAGATCCTGGAGTG	182	0.995	89
		TTTGTCGCTGTGGGACTCAG			
*agrp1*	NM_001146677.1	ATGGTCATCTCAGTATTCCCAT	152	0.9998	96
	XM_014182676.1	AGAGAGCCTTTACCGATATCTG			
	XM_014182677.1				
*agrp2*	NM_001146678.1	TGTTTCGCCGAAGACCTGAA	142	0.9986	101
		GTTTCTGAAATGCAACGTGGTG			
*mc4ra1*	XM_014140480.1	GTCATCGCCGCCATCATTAAG	152	0.9997	95
	XM_014140481.1	CCAATCCCCAGATTTCCGTC			
	XM_014140482.1				
*mc4ra2*	XM_014190362.1	TGGCAACTTGGGTATCGGC	170	0.9995	98
		GGCGCACGGTCATAATGTTG			
*mc4rb1*	XM_014157590.1	GGCGGTAATCGTGTGCATCT	185	0.9997	95
		GCACGGCGATCCTCTTTATG			
*mc4rb2*	XM_014180569.1	GAGCTCCCCGGGAAATAGTG	153	0.9996	97
		AGTGCAAATCAGTCCTCACCA			

*Primer sequences, amplicon sizes (bp), *R*^2^, and qPCR efficiency (in %) are indicated for each primer pair. *agrp*, *agouti-related protein*; *mc4r*, *melanocortin-4 receptor*; *pomc*, *proopiomelanocortin*.*

### Quantitative RT-PCR

To quantify the absolute mRNA abundance for each gene, qPCR products were purified using QIAquick PCR purification Kit (Qiagen, Hilden, Germany) and used to generate a standard curve using a 10-fold dilution series (initial concentration 10^10^ number of copies).

qPCR was carried out using 10 μl of iTaq Universal SYBR Green supermix (Bio-Rad, CA, United States), 0.6 μl of forward and 0.6 μl of reverse primers each (10 μM), 6.8 ultrapure water (Biochrom, Berlin, Germany), and 2 μl cDNA template (40 or 50 ng/reaction).

All reactions were run in duplicate, and a non-template control, no-reverse transcriptase control, and a positive between plate controls were always included. The following RT-PCR protocol was performed: (1) 95°C for 30 s, (2) 95°C for 5 s, (3) 60°C for 25 s, (4) repeating steps 2–3 for 39 more times. Melting curve analysis over a range of 65°C–95°C (increment of 0.5°C for 2 s) allowed the detection of non-specific products and/or primer dimers. The qPCR was performed using CFX96 Real-Time System (Bio-Rad Laboratories, CA, United States) in connection to CFX Manager Software version 3.1 (Bio-Rad, Laboratories, CA, United States).

Subsequently, the absolute mRNA expression level for each gene was determined based on the respective standard curve using the following equation:

$$C\,o\,p\,y\,n\,u\,m\,b\,e\,r=10^{\left(\frac{C\,q-i\,n\,t\,e\,r\,c\,e\,p\,t}{s\,l\,o\,p\,e}\right)}$$

The copy number was normalized using the total ng of RNA used for each target gene.

### Statistical Analysis

All statistical analyses were performed using GraphPad (GraphPad Software, version 8). Data related to the *K* were analyzed by two-way ANOVA followed by Sidak posttest. Equal variances and normality of distribution of gene expression were assessed using F-test and Shapiro–Wilk normality test. To achieve normal distribution, data were log-transformed and the analysis of differential expression between the fed and fasted groups was performed with two-tailed *t*-test. When either the *F*-test or the normality test failed, the no-parametric Mann–Whitney test was performed. Two-way analysis of variance (ANOVA) followed by the Sidak *post hoc* test was used to examine differences in the expression within the brain regions and the two treatment groups. A *p* < 0.05 was considered significant. All data are presented as mean ± SEM, unless otherwise stated.

## Results

### Characterization of Mc4r in Atlantic Salmon and Phylogenetic Analysis

**FIGURE 1:**
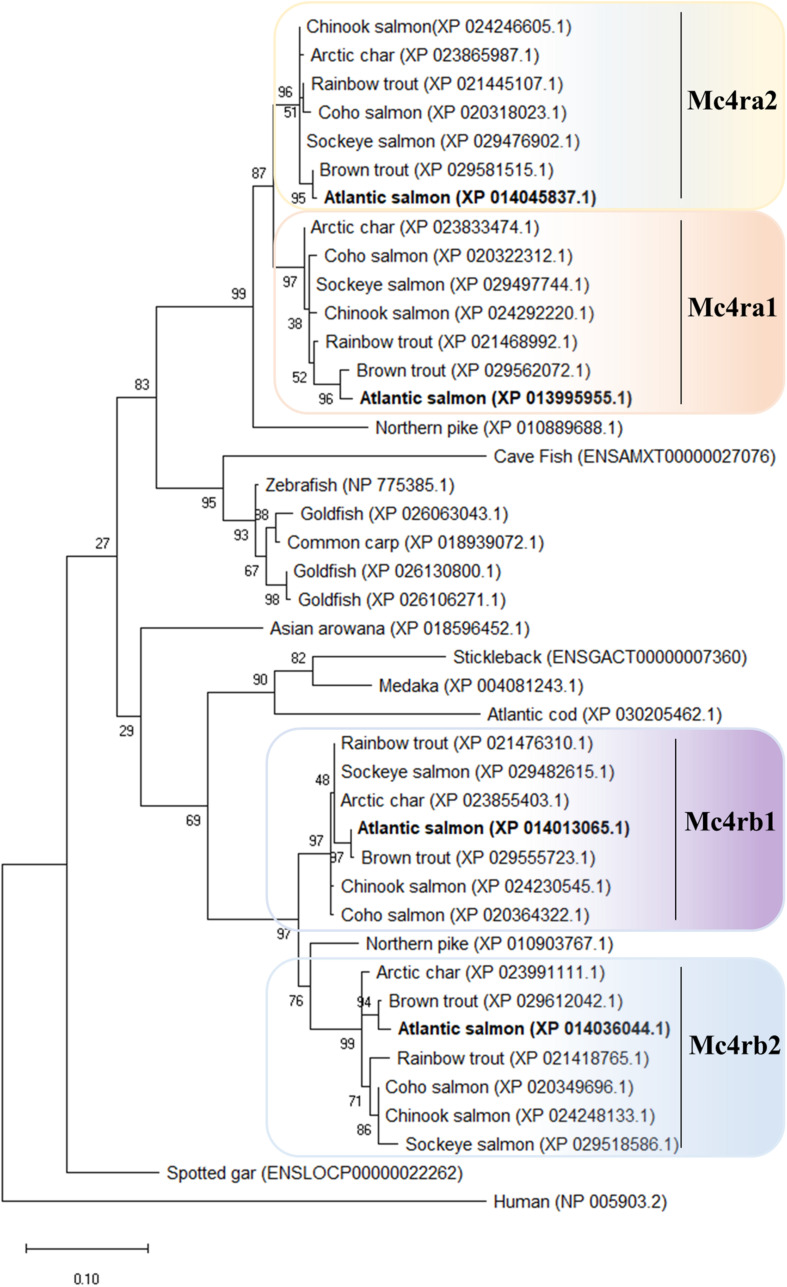
Phylogenetic relationship of melanocortin-4 receptor (Mc4r) in Salmonidae family. The phylogenetic tree was constructed based on the predicted full-length peptide sequences using the maximum likelihood (ML) method, 1,000 bootstraps replicates, and JTT + G matrix-based model in MEGA X. The tree with the highest log likelihood (–3598.40) is shown. Protein ID accession numbers are shown after the species name. The percentage of trees in which the associated taxa clustered together is shown next to the branches. Phylogenetic tree is rooted to the human MC4R sequence. For additional information related to the protein sequence alignment, please refer to Supplementary Figure 2.

**FIGURE 2:**
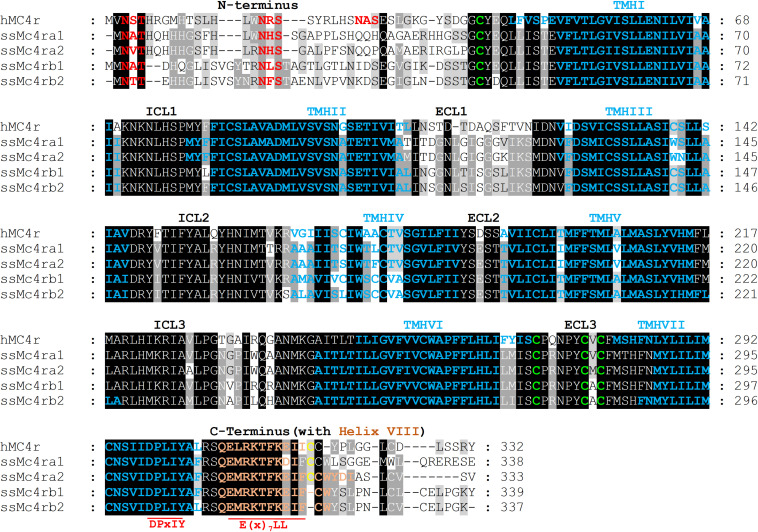
Primary protein sequence alignment of the human melanocortin-4 receptor (hMC4R) and the Atlantic salmon paralogs (ssMc4ra1, a2, b1, and b2). The transmembrane domains for hMC4R (as reviewed in UniProt) and ssMc4r (as predicted in UniProt) are marked in blue. The N-terminal, extracellular loops (ECLs) 1–3, intracellular loops (ICLs) 1 to 3, and C-terminus (with helix VIII) are also shown. The N-terminal glycosylated amino acid residues and the important conserved motifs of GPCRs are marked in red (Rodrigues et al., 2013). C-terminal palmitoylation Cys is shown in yellow. The Cys involved in the disulfide bonds in hMC4R and those conserved in ssMc4r are in green.

**FIGURE 3:**
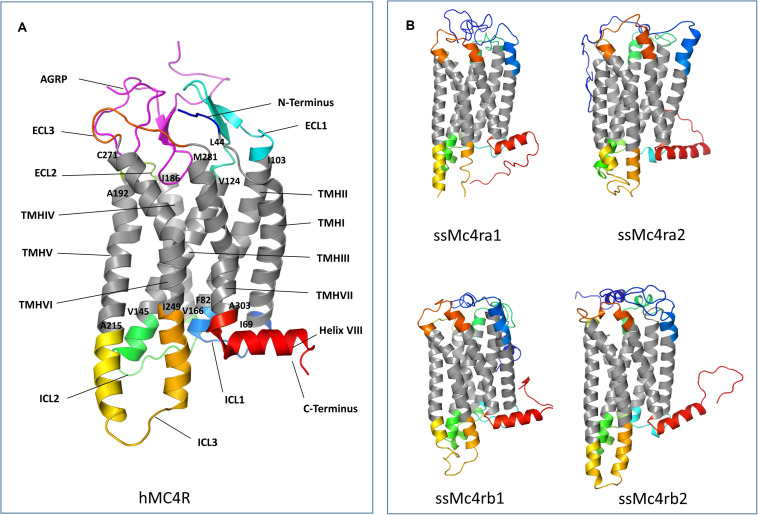
Tertiary structure of human melanocortin-4 receptor (MC4R) and Atlantic salmon Mc4r paralogs. Three-dimensional (3D) protein structures were obtained from IntFOLD and edited in the PyMOL Molecular Graphics System, Version 2.0 Schrödinger, LLC. **(A)** Human MC4R 3D structure with agouti-related protein (AGRP) (PDB entry 2IQV). **(B)** Atlantic salmon Mc4r paralogs 3D structure. N-terminal domains are colored in dark blue, C-terminal domains in red, the seven transmembrane helices (TMH) in gray. The extracellular (ECL) and intracellular (ICL) loops are represented by different colors. The boundary amino acids of TMH are labeled according to the protein sequence alignment.

In salmonids, four Mc4r protein paralogs (Supplementary Figure 2) were identified, showing well-conserved domains with respect to other ortholog sequences within teleost. In Atlantic salmon, Mc4r paralogs were found to be encoded by genes located on chromosomes ss03 (Mc4ra2), ssa14 (Mc4ra1), ssa19 (Mc4rb1), and ssa29 (Mc4rb2). The predicted AA sequence of Atlantic salmon Mc4r varied from 333 to 339 AA in length, and protein weighed from 37.37 to 37.99 kDa (data retrieved from UniProt). The paralogs Mc4ra1 and a2, and paralogs Mc4rb1 and b2, shared 89% identity at the AA level, whereas Mc4ra and Mc4rb shared at least 73% of identity. All four paralogs are relatively well-conserved with the human homolog, sharing from 63 to 68% of AA sequence identity. Atlantic salmon Mc4r paralogs shared from 73 to 90% AA identity with northern pike Mc4r and 73 to 95% of identity with other salmonid species (Supplementary Table 3). In the phylogenetic analysis, the teleost Mc4r divided into two clades and the Atlantic salmon Mc4r paralogs clustered into four different groups (Figure 1), each containing species belonging to the Salmonidae family. Each cluster, except for the Mc4rb1, branches from the northern pike. According to our phylogenetic analysis, two Mc4r duplicates (Mc4ra and Mc4rb) are present in salmonids and northern pike. In addition, salmonids have two copies of Mc4ra (Mc4ra1, Mc4ra2) and two copies of Mc4rb (Mc4rb1 and Mc4rb2) possibly as a result of the Ss 4R WGD. The alignment of human MC4R and Atlantic salmon Mc4r showed well-conserved seven transmembrane domains with divergent AA residues at N-terminus, ECL1, and C-terminus (Figures 2, 3). Two of three N-terminal asparagine (N) N-glycosylated sites (NxS/T) are also conserved in Atlantic salmon (Figure 2). Further, the C-terminal palmitoylation site cysteine (Cys) residue Cys318 in human MC4R is conserved in the Atlantic salmon Mc4ra1 and a2. Importantly, motif DPxIY and C-terminal motif E(x)_7_LL for G Protein-Coupled Receptors (GPCRs) [reviewed in Rodrigues et al. (2013)] are conserved in all salmon paralogs. There are two putative disulfide bonds in human MC4R, one between Cys271 (TMHVI) and Cys277 (ECL3) and another between Cys40 (N-terminus) and Cys271 (ECL3). Our predicted model showed one disulfide bond within the ECL3 for Mc4ra1 (Cys274 and Cys280) and Mc4rb2 (Cys275 and Cys281) in Atlantic salmon (Supplementary Figure 5).

### Characterization of Mature Pomc in Atlantic Salmon and Phylogenetic Analysis

In Atlantic salmon, three previously identified Pomc protein paralogs were located in chromosomes ssa01 (Pomca2), ssa06 (Pomcb), and ssa09 (Pomca1). The predicted AA sequence length of Atlantic salmon Pomc varied from 225 to 232 AA and protein weight from 24.7 to 25.9 kDa (data retrieved from UniProt). Pomca1 and a2 shared 84% of AA identity (Supplementary Table 4). Pomcb AA sequence shared 36% of identity with Pomca1 and a2. Salmon Pomc paralogs shared 27–37% AA sequence identity with the human Pomc and 31–51% with northern pike Pomc. Atlantic salmon Pomc shared from 32 to 98% of AA identity with other species from the Salmonidae family. Atlantic salmon Pomc, as the human homolog, has a signal peptide of 26 AA, with the exception of Pomcb, which has a 21-AA signal peptide (Supplementary Figure 3). As expected, the phylogenetic analyses divided the salmonid Pomc peptide sequences in two main clusters Pomca and Pomcb (Supplementary Figure 4). The salmonid Pomca and Pomcb clustered with the northern pike and the Neoteleostei Pomca and Pomcb sequences, respectively. The salmonids have two copies of Pomca, whereas common carp and goldfish have duplicate Pomcs that belong to each of two separate clades. The phylogenetic tree suggests that the duplicated *pomc* would have evolved from Ts WGD.

The predicted posttranslational cleavages sites in Atlantic salmon were determined taking a comparative homology approach using the human homolog protein (Supplementary Figure 4). The human KR, KRR, and KK cleavage sites lead Pomc into mature peptide hormones: α-, β-, and γ-MSH, ACTH, corticotropin-like intermediate peptide (CLIP), β- and γ-LPH (lipotropin), β-endorphin, and INN (Met-enkephalin). In the teleost species analyzed, the same potential KR, KK, and RR cleavage sites were present in Pomca1 and a2, while in Pomcb, the last KK cleavage site was not present. Moreover, the alignment confirmed the lack of γ-MSH in teleost compared to human.

### Brain Distribution of Atlantic Salmon *mc4r*, *pomc*, and *agrp* mRNA

**FIGURE 4:**
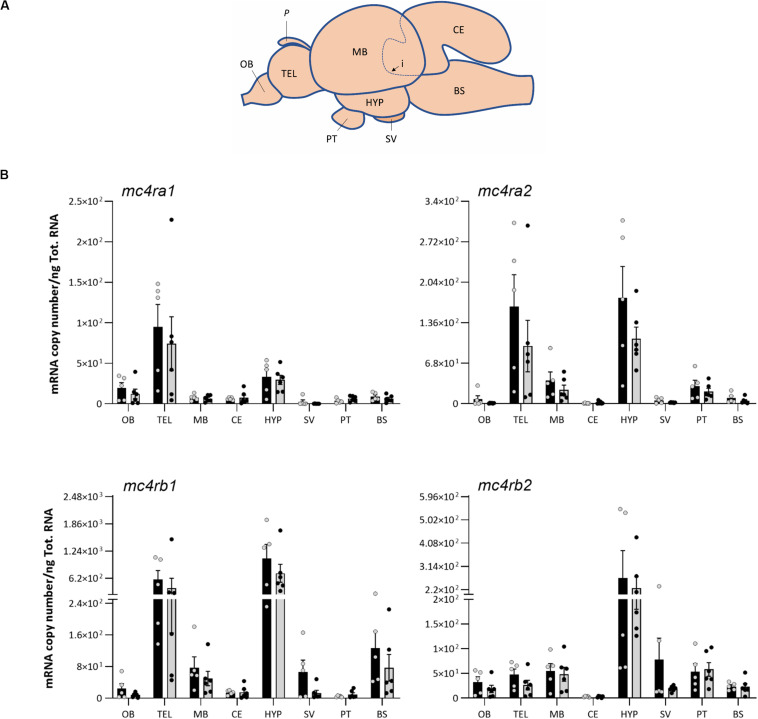
**(A)** Schematic representation of Atlantic salmon post smolt brain, showing dissection of the eight brain regions for gene expression analysis: olfactory bulb (OB), telencephalon (TEL), midbrain (MB), hypothalamus (HYP), cerebellum (CE), saccus vasculosus (SV), pituitary (PT), and brain stem (BS). Dashed line (i) represents the dissected area of the CE inside the MB, and P represents the pineal gland. **(B)** Effects of 4 days of fasting on the mRNA expression levels of *melanocortin-4 receptor* (*mc4r*) paralog genes in eight regions of Atlantic salmon brain. Black and gray columns represent fed (*n* = 6) and fasted (*n* = 6) fish, respectively. Values are expressed as copy number per total RNA used in the reaction. The dots represent the individual fish, and bars represent mean ± SEM. Two-tailed *t*-test was performed to assess the statistically significant differences between the two groups. Interaction between the brain region response and the treatment was analyzed with two-way ANOVA (Supplementary Table 1 for detailed information).

**FIGURE 5:**
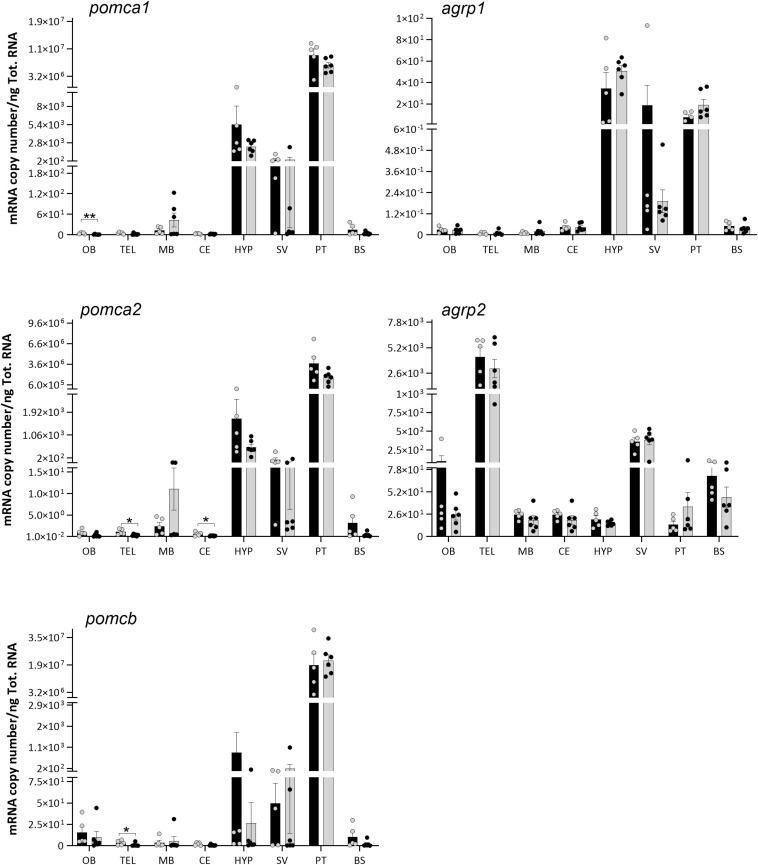
Effects of 4 days of fasting on the mRNA expression levels of *proopiomelanocortin* (*pomc*) and *agouti-related protein* (*agrp*) paralog genes in eight regions of Atlantic salmon brain. Black and gray columns represent fed (*n* = 6) and fasted (*n* = 6) fish, respectively. Values are expressed as copy number per total RNA used in the reaction. The dots represent individual fish, bars represent mean ± SEM, and asterisks show the significant degree (*p < 0.05, **p < 0.01). Two-tailed *t*-test was performed to assess the statistically significant differences between the two groups. Interaction between the brain region response and the treatment was analyzed with two-way ANOVA, followed by Sidak posttest (refer to Supplementary Table 1 for detailed information).

Both the melanocortin receptor *mc4r* and the neuropeptides *pomc* and *agrp* mRNA analyzed in this study showed a wide distribution in the eight brain regions (Figures 4, 5). All Atlantic salmon *mc4r* genes showed high mRNA expression levels in the hypothalamus, whereas *mc4ra1* was more abundant in the telencephalon (Figure 4). Interestingly, *mc4ra2* and *mc4rb1* showed a predominant mRNA abundance in the telencephalon and hypothalamus, and *mc4rb2* was high in the hypothalamus and similar expression level in other regions. The *mc4rb1* was the most abundant paralog in the Atlantic salmon brain. All *mc4r* paralogs show low mRNA expression levels in the olfactory bulb, cerebellum, and saccus vasculosus.

The *pomca1*, *pomca2*, and *pomcb* were predominantly expressed in the pituitary, followed by hypothalamus and saccus vasculosus, but low levels of expression were also found in other brain regions (Figure 5). *Agrp1* showed a prevalence gene expression in the hypothalamus, pituitary, and saccus vasculosus (Figure 5). On the contrary, *agrp2* was mainly expressed in the telencephalon, saccus vasculosus, and olfactory bulb.

### Effects of 4 Days of Fasting in Atlantic Salmon

The *K* factor of fed and fasted Atlantic salmon was significantly different (two-way ANOVA *p* = 0.0010) (Supplementary Figure 1). After 4 days of fasting, fish showed a significantly lower *K* factor (1.035 ± 0.011) than the fed group (1.095 ± 0.015) (Sidak posttest *p* = 0.0027) (Supplementary Table 1).

No significant differences in the mRNA expressions of *mc4r*, *pomc*, and *agrp* paralogs were observed between the fed and fasted groups (Figures 4, 5) in any highly expressed brain regions, i.e., hypothalamus, telencephalon, pituitary, and saccus vasculosus. On the other side, 4 days of fasting had a significant effect on the expression of *pomca1*, *pomca2*, and *pomcb* (Figure 5) in very low expressed regions (Supplementary Table 2). A significant decrease was found in the expression of *pomca1* in the olfactory bulb (*t*-test *p* = 0.0057), of *pomca2* in the telencephalon (*t*-test *p* = 0.0233) and cerebellum (*t*-test *p* = 0.0340), and on *pomcb* in the telencephalon (Mann–Whitney *p* = 0.0303). *pomca1* showed a decreased tendency in the telencephalon of the fasted group, although not statistically significant (*t*-test *p* = 0.0873). Both *agrp1* and a*grp2* did not show any significant difference between the fed and fasted groups (Figure 5). A high individual variation was observed in the mRNA expression levels of the target genes analyzed.

## Discussion

The role of the melanocortin system on appetite and energy homeostasis in Atlantic salmon is still poorly understood. In addition, the presence of several paralog genes as a result of the Ss 4R WGD has led to possible divergent functions of the key genes involved in this system.

In the current study, we report for the first time the identification and characterization of the Atlantic salmon receptor Mc4r. Our *in silico* analysis revealed the presence of four paralog genes *mc4ra1*, *mc4ra2*, *mc4rb1*, and *mc4rb2*, clustered into four different groups in all salmonid species analyzed. The presence of four Mc4r in Atlantic salmon (and other salmonids) appears to be the result of the Ss 4R WGD. The presence of *mc4ra* and *mc4rb* homologs in Northern pike suggests that the origin of these genes occurred either just prior to the divergence between Salmoniformes and Esociformes or it is a result of an independent species-specific duplication. Cypriniformes, such as common carp and goldfish, have also experienced additional 4RWGD around 50–16 mya subsequent to the Ts WGD and Ss 4R WGD. Consequently, we found three Mc4r in goldfish but only one Mc4r in common carp (Larhammar and Risinger, 1994; David et al., 2003). Moreover, the Cypriniformes Mc4r is distantly related to the other analyzed teleost, which is reflected in the separated clade and seems to be the Mc4ra type. The confirmed mRNA sequences of the qPCR amplicons indicated that all four salmon *mc4r* genes are not pseudogenes. The Atlantic salmon showed well-conserved seven hydrophobic transmembrane domains as well as one putative disulfide bond, within the ECL3 for Mc4ra1 (Cys274 and Cys280) and Mc4rb2 (Cys275 and Cys281), as described for human MC4R (Cys271 and Cys277) (Chai et al., 2005; Chapman et al., 2010; Heyder et al., 2019). Even though Cys are also present in the primary sequence of Mc4ra2 and Mc4rb1, a disulfide bond was not present in the predicted tertiary structure in PyMOL. Natural mutation occurring in the human Cys271 (C271R and C271Y) have been linked to severe MC4R functional changes, but this AA substitution was not found in the predicted Atlantic salmon Mc4r sequence. Further analysis is needed to investigate these aspects in this species. The human N-glycosylated site (NxS/T) located in the N-terminus is also present in Atlantic salmon Mc4r paralogs. The glycosylated site and the disulfide bonds are important for the receptor structure folding, stability, and target trafficking (Chai et al., 2005; Tao, 2010; Rodrigues et al., 2013). Furthermore, the palmitoylation site at the Cys residue Cys318 of the C-terminus in human MC4R is also present in the Atlantic salmon Mc4ra1 and a2. The conserved C-terminal Cys318 serving as palmitoylation site might possibly lead to a fourth intracellular loop by anchoring the C-terminus to the cell membrane [reviewed in Tao (2010)]. Importantly, GPCR motifs N/DPxIY and E(x)_7_LL [reviewed in Rodrigues et al. (2013)] are also present in all Mc4r salmon paralogs. The N/DPxIY motif acts as an on/off switch with two conformational changes according to the active and inactive states (Chapman et al., 2010; Rodrigues et al., 2013), whereas E(x)_7_LL motif seems to be important in anterograde trafficking of MCRs (from endoplasmic reticulum to cell surface) [reviewed by Rodrigues et al. (2013)].

In general, for human MC4Rs, the pocket of aspartic acid Asp122/126 in TMHIII and basic histidine (His) 264 residues in TMHVI (Metz et al., 2006; Chapman et al., 2010; Wen et al., 2017; Heyder et al., 2019) along with ECL2 and ECL3 (Tao, 2010) are essential for ligand binding. β-MSH has been shown to the have the highest affinity to human MC4R, followed by α-MSH and ACTH (Tao, 2010). The same pocket seems to be conserved in Atlantic salmon; however, future studies are necessary to explore the ligand–Mc4r interactions in this species.

In the mammalian hypothalamus, numerous interconnecting nuclei, as the arcuate nucleus (ARC), ventromedial nucleus (VMN), dorsomedial nucleus (DMN), paraventricular nucleus (PVN), and lateral hypothalamus (LH), are organized into a complex neuronal network that plays a crucial role in the central control of appetite (Bouret et al., 2004; Rønnestad et al., 2017; Soengas et al., 2018). The ARC has been described as the location for neurons expressing POMC and AGRP that project to the hypothalamic PVN where the MC4R is located (Ghamari-Langroudi et al., 2011; Hall, 2011). In teleost, the lateral tuberal nucleus (NLT) in the hypothalamus has been described as the homolog of the mammalian ARC (Cerdá-Reverter and Peter, 2003; Cerdá-Reverter et al., 2003a, b). In goldfish and spotted sea bass, *in situ* hybridization showed neurons expressing *mc4r* in the telencephalon, thalamus, preoptic area, and hypothalamus (NLT and hypothalamic inferior lobe) (Cerdá-Reverter et al., 2003a; Zhang et al., 2019). Similarly, *agrp* and *pomc* were found in the NLT and in rostral hypothalamus of gold fish and rainbow trout (Cerdá-Reverter and Peter, 2003; Cerdá-Reverter et al., 2003b; Otero-Rodiño et al., 2019).

Our results showed that all the Atlantic salmon *mc4r* paralogs were predominantly expressed in the hypothalamus and telencephalon, even though to a lesser extent expressed in other regions of the brain. It seems therefore that the hypothalamus and telencephalon are the major functional sites for the central *mc4r* in Atlantic salmon, but their role in appetite regulation is still unclear. These results are in line with the study of Zhang et al. (2019), where high *mc4r* levels were detected in the telencephalon and diencephalon of spotted sea bass. Among the Atlantic salmon *mc4r* paralogs, *mc4rb1* had the highest levels of expression, particularly in the hypothalamus, but its role in appetite regulation it is still unclear.

In this study, we have extended the current knowledge on Atlantic salmon *pomc* and *agrp* on appetite regulation, which was previously based on the analysis of the whole brain (Murashita et al., 2009, 2011) or on the hypothalamus (Kalananthan et al., 2020). Murashita et al. (2011, 2009) identified and characterized three *pomc* gene paralogs (*pomca1*, *a2*, and *b*) and one splice variant (*pomca2s*) and two *agrp* paralogs (*agrp1* and *2*). In goldfish, *in situ* hybridization studies demonstrated *pomc* mRNA cell bodies exclusively expressed within the mediobasal hypothalamus, in the NLT, and in the medial region of the lateral recess nucleus (Cerdá-Reverter et al., 2003b). In our spatial analysis, we found a clear dominant expression of *pomca1, pomca2*, and *pomcb* in the pituitary, followed by the hypothalamus and saccus vasculosus. The pituitary is an important site of *pomc* expression, where it is further post-translated into ACTH and α-MSH, responsible for the biosynthesis of glucocorticoids (e.g., cortisol) from the adrenal cortex (Dunn and Berridge, 1990). On the other hand, α-MSH in the hypothalamus activates the MC4R, leading to reduced food intake and increased energy expenditure (Anderson et al., 2016). The α-MSH has been described as the most well-conserved posttranslated forms of Pomc, underlining the strong functional constraint along the vertebrate lineage (Takahashi and Kawauchi, 2006).

The *agrp* paralogs showed different spatial distributions in the brain of Atlantic salmon. The *agrp1* was mainly expressed in the hypothalamus, as also described in previous studies in the ventral neurons of the NLT and rostral hypothalamus in goldfish (Cerdá-Reverter and Peter, 2003; Cerdá-Reverter et al., 2003a), sea bream (Koch et al., 2019), and rainbow trout (Otero-Rodiño et al., 2019). The *agrp1* mRNA was also detected in other regions of the brain as saccus vasculosus and pituitary. *Agrp2* showed high expression levels in telencephalon and saccus vasculosus. Recently, Agrp1 was reported to be involved in the control of food consumption in zebrafish, while the Agrp2 in the preoptic neurons was suggested to act as a neuroendocrine regulator of stress response by downregulating cortisol secretion (Shainer et al., 2019).

The role of AGRP, POMC, and MC4R on appetite regulation have been suggested to be evolutionarily conserved across vertebrates (Ghamari-Langroudi et al., 2011; Kim et al., 2014; Nuzzaci et al., 2015). However, in the present study, no significant differences in mRNA expression of *mc4r*, *agrp*, or *pomc* paralogs between fed and fasted states were observed in the hypothalamus, which has been described as the central area in the control of appetite in mammals and teleost fishes (Nuzzaci et al., 2015; Volkoff, 2016; Rønnestad et al., 2017). Similar results were also observed in sea bass, where 4 days of food deprivation did not affect the *mc4r* expression in the hypothalamus or *pomc* mRNA expression in the hypothalamus and pituitary (Sánchez et al., 2009). Few studies have described that Mc4r was downregulated when feeding was restricted in teleost species (Wan et al., 2012; Aspiras et al., 2015; Striberny et al., 2015; Zhang et al., 2019). In barfin flounder, in contrast, the *mc4r* was downregulated in the liver, but no changes in expression were detected in the brain by fasting (Kobayashi et al., 2008). However, a previous study in sea bass showed the Mc4r activity to be dependent on the Agrp binding rather than the *mc4*r expression in case of progressive fasting (Sánchez et al., 2009). The importance of Mc4r in the regulation of appetite in fish is emphasized by naturally occurring mutations in Mexican cavefish (Aspiras et al., 2015). In this species, coding mutations in conserved residues reduces the signaling efficiency and basal activity of the Mc4r probably due to adaptation to long-term starvation and sporadic food availability (Aspiras et al., 2015).

On the other hand, 4 days of fasting had a significant effect on the expression of *pomca1*, *pomca2*, and *pomcb* in other regions of the Atlantic salmon brain. A significant decrease was found at the mRNA level of *pomca1* in the olfactory bulb, *pomca2* in the telencephalon and cerebellum, and *pomcb* in the telencephalon. However, it is important to underline that *pomc* was very lowly expressed in these regions, and it is not yet clear if these brain regions actually contribute to the appetite regulation in Atlantic salmon. In coho salmon, the posttranslational Pomc*-*derived α-Msh exhibits an anorexigenic effect (White et al., 2016). These authors showed that intraperitoneal injections of α-Msh suppressed feed intake, acting as an anorexigenic factor. However, in rainbow trout, 14 days of fasting did not have any effect on the mRNA expression of the three *pomc* paralogs (*pomca1*, *a2*, and *b*), but 28 days of fasting favored the decrease in hypothalamic *pomca1*, but not in *pomca2* or *pomcb* (Leder and Silverstein, 2006). Furthermore, hyperglycemic conditions increased the hypothalamic *pomca1* mRNA expression levels in rainbow trout (Conde-Sieira et al., 2010; Otero-Rodiño et al., 2015). In the whole-brain analysis of Atlantic salmon, the upregulation of *pomca1* (3 h post-feeding) and *pomcb* (0.5 and 6 h post-feeding) was suggested to represent a role in short-term feeding regulation (Valen et al., 2011).

In teleost, Agrp shows different functions depending on the region of the brain where it is expressed. In zebrafish, hypothalamic *agrp1* was proposed to have a similar function in the control of appetite and food intake as in mammals, whereas *agrp2* in the preoptic region acted as a stress regulator (Shainer et al., 2017, 2019). These authors also found *agrp2* to be expressed in the pineal and proposed that it may have a novel function rather than a neuroendocrine role involved in the regulation of the stress axis. An upregulation of Agrp was observed in short-term fasting of early larvae of Ya-fish (Wei et al., 2013). This was supported by Song et al. (2003) in zebrafish and Koch et al. (2019) in gilthead sea bream larvae, where starving increased *agrp1* expression. In another study, Agulleiro et al. (2014) reported the involvement of both *agrp* paralogs in appetite regulation by showing an increase of hypothalamic *agrp1* and decrease of *agrp2* in sea bass when subject to progressive fasting. In rainbow trout, Comesaña et al. (2017) showed that ICV of leucine decreased feed intake with a decrease in mRNA abundance of *agrp*. In Atlantic salmon, Murashita et al. (2009) reported that whole-brain *agrp1* was downregulated after 6 days of fasting, while *agrp2* was not affected, indicating that *agrp2* may not play a role on the control of appetite. A similar effect was described in common carp (Wan et al., 2012). In contrast, in our recent study, hypothalamic *agrp1* was upregulated after 3 days of fasting in Atlantic salmon (Kalananthan et al., 2020). However, in the current study, no differences were observed in the hypothalamus or other regions between the fed and fasted groups for either *agrp1* or *agrp2*.

The identification and basic characterization of the multiple paralogs of the appetite-regulating genes provide an essential groundwork to elucidate their functional role in the central control of food intake in Atlantic salmon. Minimal or no effects of fasting on the mRNA expression of the investigated genes suggest that they play a minor role in the central control of appetite in the short 4 days’ fasting. However, a high individual variability was observed in both fed and fasted experimental groups, which might have led to a possible deviation in the results from our recent study on hypothalamic *agrp1* in Atlantic salmon (Kalananthan et al., 2020). Among the factors that contribute to the differences in feed intake, the feeding rate, frequency and time, and social relationships between conspecifics are the result of stimulating competition for a feed resource among individuals (Attia et al., 2012). Differences in physiology, life stages, feeding requirements, living environments, and individual variability might be at the base of the species-specific responses (Volkoff, 2016; Soengas et al., 2018). In salmonids as in other teleost species, the presence of dominant individuals can increase aggression and inhibition and limit food viability toward subordinate fishes, leading to differences in feeding behavior (Gilmour et al., 2005). Moreover, the difference in the sampling protocols and methodology used increases the complexity and variability of the data when comparing across and in between species.

The genome of teleost, compared to mammals, is the result of a third round (3R) or 4R (e.g., Salmonidae) of WGD. This evolutionary duplication make the teleost a potentially more complex model to study the function of feed-regulating factors in comparison to the mammalian homologs (Volkoff, 2016). This fact should be taken into consideration when comparing studies in teleost *versus* mammals.

In conclusion, the present study provides new understanding of the still limited information available on the appetite regulation in Atlantic salmon. The identification of the multiple paralogs of *mc4r*, *pomc*, and *agrp* and their wide distribution in Atlantic salmon brain provide novel insights and lay the groundwork for experimental studies. Fasting did not affect the mRNA expression levels of melanocortin system players in the hypothalamus compared with fed fish. Further studies exploring the mRNA and/or protein localization within the brain areas and functional characterization are needed to elucidate the role of the melanocortin system in the central control of food intake in Atlantic salmon.

## Data Availability Statement

All datasets generated for this study are included in the article/Supplementary Material.

## Ethics Statement

The animal study was reviewed and approved by local representative of Animal Welfare (at Department of Biological Sciences, University of Bergen, Norway) in accordance with Norwegian Animal Research Authority regulations.

## Author Contributions

All authors conceived and designed the study, contributed to the writing of the manuscript, and read and approved the submitted version. TK, SH, IR, and FL contributed to the sampling. KM, FL, and AG did the preparatory lab work. TK and FL performed the qPCR analysis. IR made the schematic illustration of Atlantic salmon brain. TK performed the tertiary protein structures. FL did the statistical and phylogeny analyses.

## Conflict of Interest

The authors declare that the research was conducted in the absence of any commercial or financial relationships that could be construed as a potential conflict of interest.

## Footnotes

1 https://www.ncbi.nlm.nih.gov/

2 http://www.ensembl.org/index.html

3 https://www.uniprot.org/

4 http://www.cbs.dtu.dk/services/ProP/

5 https://pymol.org/2/

6 http://primer3.ut.ee/
