# Supplementary material for: The Melanocortin System in Atlantic Salmon (Salmo salar L.) and Its Role in Appetite Control
Source: Front Neuroanat. 2020 Aug 21;14:48. doi: 10.3389/fnana.2020.00048 (PMC7471746; doi:10.3389/fnana.2020.00048)
Supplement: Supplementary file 1 [file Presentation_1.pdf]

Supplementary Material

The Melanocortin System in Atlantic salmon (*Salmo salar* L.) and its Role in Appetite Control

Tharmini Kalananthan[1]*, Floriana Lai[1]*, Ana S. Gomes[1], Koji Murashita[1,2], Sigurd Handeland[1,3], Ivar Rønnestad[1]

[1] Department of Biological Sciences, University of Bergen, Bergen, Norway
[2] Research Center for Aquaculture Systems, National Research Institute of Aquaculture, Japan Fisheries Research and Education Agency, Tamaki, Japan
[3] Norwegian Research Center, NORCE Environment, Bergen, Norway

*Authorship equal

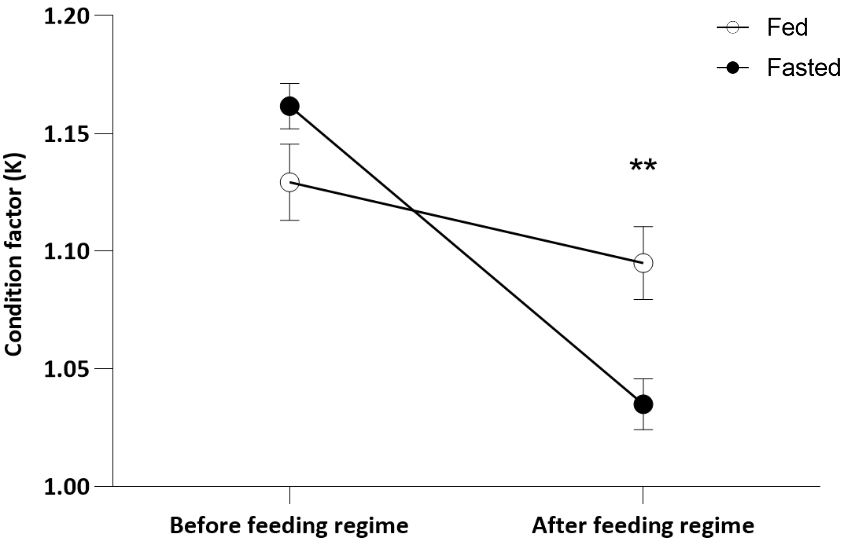

Supplementary Figure 1. Condition factor (*K*) before and after the 4 days of fasting. Data was analysed by two-way ANOVA followed by Sidak post-test. White dots represent Atlantic salmon before (*n*=21) and after (*n*=27) 4 days fed *ad libitum*. Black dots represent Atlantic salmon before (*n*=21) and after (*n*=26) 4 days of fasting. All data are presented as mean ± SEM.

```
Arctic char(XP_023855403.1):-----MNST--DHQGLISVGYTRNLSTAGTLGTLNKDSEGVGIKDSSTGCYEQLLISTEVFLTLGIVSLLENILVIAAIIKNKNLHSPMYLFICSLAVADMLVSVSNASE : 103
Rainbow trout(XP_021476310.1):--MMNST--DHQGLISVGYTRNLSTAGTLGTLNKDSEGVGIKDSSTGCYEQLLISTEVFLTLGIVSLLENILVIAAIIKNKNLHSPMYLFICSLAVADMLVSVSNASE : 104
Sockeye salmon(XP_029482615.1):-MMNST--DHQGLISVDYTRNLSTAGTLGTLNKDSEGVGIKDSSTGCYEQLLISTEVFLTLGIVSLLENILVIAAIIKNKNLHSPMYLFICSLAVADMLVSVSNASE : 104
Coho salmon(XP_020364322.1):----MMNST--DHQGLISVGYTRNISTAGTLGTLNKDSEGVGIKDSSTGCYEQLLISTEVFLTLGIVSLLENILVIAAIIKNKNLHSPMYLFICSLAVADMLVSVSNASE : 104
Chinook salmon(XP_024230545.1):-MMNST--DHQGLISVGYTRNLSTAGTLGTLNKDSEGVGIKDSSTGCYEQLLISTEVFLTLGIVSLLENILVIAAIIKNKNLHSPMYLFICSLAVADMLVSVSNASE : 104
Atlantic salmon(XP_014013065.1):MMNAT--DHQGLISVGYTRNLSTAGTLGTLNIDSEGVGIKDSSTGCYEQLLISTEVFLTLGIVSLLENILVIAAIIKNKNLHSPMYLFICSLAVADMLVSVSNASE : 104
Brown trout(XP_029555723.1):----MMNAT--DHQGLISAGYTRNLSTAGTLGTLNIDSEGVGIKDSSTGCYEQLLISTEVFLTLGIVSLLENILVIAAIIKNKNLHSPMYLFICSLAVADMLVSVSNASE : 104
Brown trout(XP_029612042.1):-----MNAT--EHHGLISVSYNRNSSTAGNLVPVNKDSEGIGLNDSSTGCYDQLLISTEVFLTLGIVSLLENILVIAAIIKNKNLHSPMYFFICSLAVADMLVSVSNASE : 103
Atlantic salmon(XP_014036044.1):-MNTT--EHHGLISVSYNRNFSTAENLVPVNKDSEGIGLNDSSTGCYDQLLISTEVFLTLGIVSLLENILVIAAIIKNKNLHSPMYFFICSLAVADMLVSVSNASE : 103
Chinook salmon(XP_024248133.1):--MNAT--EHHGLISVSYNRNSSTAGSLVSINKDSEGIGLNDSSTGCYDQLLISTEVFLTLGIVSLLENILVIAAIIKNKNLHSPMYFFICSLAVADMLVSVSNASE : 103
Coho salmon(XP_020349696.1):-----MNAT--EHHGLISVSYNRNSSTAGSLVSVNKDSEGIGLNDSSTGCYDQLLISTEVFLTLGIVSLLENILVIAAIIKNKNLHSPMYFFICSLAVADMLVSVSNASE : 103
Sockeye salmon(XP_029518586.1):--MNVT--EHHGLISVSFNGNSSTAGSLVSVNKDTEGIGLNDSSTGCYDQLLISTEVFLTLGIVSLLENILVIAAIIKNKNLHSPMYFFICSLAVADMLVSVSNASE : 103
Arctic char(XP_023991111.1):-----MNAT--EHHGLISVSYNRNSSTAGTLVSVNKDSEGIGLNDSSTGCYDQLLISTEVFLTLGIVSLLENILVIAAIIKNKNLHSPMYFFICSLAVADMLVSVSNASE : 103
Rainbow trout(XP_021418765.1):---MNAT--EYQGSISVSYNRNSSTAGTLVSVNKDSEGIGLNDSSTGCYDQLLISTEVFLTLGIVSLLENILVIAAIIKNKNLHSPMYFFICSLAVADMLVSVSNASE : 103
Northern pike(XP_010903767.1):--MMNVT--EHHRFISVSYNSNLSTAETLRHVNKVS---GLNDSSTGCYEQLLISTEVFLTLGIVSLLENILVIAAIIKNKNLHSPMYFFICSLAVADMLVSVSNASE : 101
Chinook salmon(XP_024246605.1):--MNATHQHHHGSFHL---RNHSSGALPFSNQQPQAMAERFRGLPGCYEQLLISTEVFLTLGIVSLLENILVIAAIIKNKNLHSPMYFFICSLAVADMLVSVSNATE : 102
Sockeye salmon(XP_029476902.1):--MNATHQHHHGSFHL---RNHSSGALPFSNQQPQAMAERFRGLPGCYEQLLISTEVFLTLGIVSLLENILVIAAIIKNKNLHSPMYFFICSLAVADMLVSVSNATE : 102
Rainbow trout(XP_021445107.1):---MNATHQHHHGSFHL---RNHSSGALPFSNQQPQAMAERFRGLPGCYEQLLISTEVFLTLGIVSLLENILVIAAIIKNKNLHSPMYFFICSLAVADMLVSVSNATE : 102
Coho salmon(XP_020318023.1):-----MNATHQHHHGSFHL---RNHSSGALPFSNQQPQAMAERFRGLPGCYEQLLISTEVFLTLGIVSLLENILVIAAIIKNKNLHSPMYFFICSLAVADMLVSVSNATE : 102
Arctic char(XP_023865987.1):-----MNATHQHHHGSFHL---RNHSSGALPFSNQQPQAMAERIRGLPGCYEQLLISTEVFLTLGIVSLLENILVIAAIIKNKNXHSPMYFFICSLAVADMLVSVSNATE : 102
Brown trout(XP_029581515.1):-----MNVTHQHHHGSFHL---RNHSSGALPFSNQQPQAMAERIRGLPGCYEQLLISTEVFLTLGIVSLLENILVIAAIIKNKNLHSPMYFFICSLAVADMLVSVSNATE : 102
Atlantic salmon(XP_014045837.1):-MNVTHQHHHGSFHL---RNHSSGALPFSNQQPQAMAERIRGLPGCYEQLLISTEVFLTLGIISLLENILVIAAIIKNKNLHSPMYFFICSLAVADMLVSVSNATE : 102
Brown trout(XP_029562072.1):-----MNATHQHHHGSFHL---WNHSSGATPLSHQQHQAGAERHHGSSGCYEQLLISTEVFLTLGIVSLLENILVIAAIIKNKNLHSPMYFFICSLAMADMLVSVSNATE : 102
Atlantic salmon(XP_013995955.1):-MNATHQHHHGSFHL---WNHSSGAPPLSHQQHQAGAERHHGSSGCYEQLLISTEVFLTLGIVSLLENILVIAAIIKNKNLHSPMYFFICSLAMADMLVSVSNATE : 102
Rainbow trout(XP_021468992.1):---MNATHQHHHGSFHL---WNHSSGAPPLSHQQHQAGAERHHGSSGCYEQLLISTEVFLTLGIVSLLENILVIAAIIKNKNLHSPMYFFICSLAMADMLVSVSNATE : 102
Coho salmon(XP_020322312.1):-----MNATHQHHHGSFHL---WNHSSGAPPLSHQQHQAGAERHHGSSGCYEQLLISTEVFLTLGIVSLLENILVIAAIIKNKNLHSPMYFFICSLAMADMLVSVSNATE : 102
Sockeye salmon(XP_029497744.1):--MNATHQHHHGSFHL---WNHSSGAPPLSHQQHQAGAERHHGSSGCYEQLLISTEVFLTLGIVSLLENILVIAAIIKNKNLHSPMYFFICSLAMADMLVSVSNATE : 102
Chinook salmon(XP_024292220.1):--MNATYQHHHGSFHL---WNHSSGAPPLSHQQHQAGAERHHGSSGCYEQLLISTEVFLTLGIVSLLENILVIAAIIKNKNLHSPMYFFICSLAMADMLVSVSNATE : 102
Arctic char(XP_023833474.1):-----MNATHQHHHGSFHL---WNHSSGAPPLSHQRHQAGAERHHGSSGCYEQLLISTEVFLTLGIVSLLENILVIAAIIKNKNLHSPMYFFICSLAMADMLVSVSNATE : 102
Northern pike(XP_010889688.1):---MNTTRQHHNGSFHL---RNHSSGALSLRHQQPPTGADRHHASTGCYEQLLISTEVFLMLGIFSLLENILVIAAIVKNKNLHSPMYFFICSLAVADMLVSVSNATE : 102
Goldfish(XP_026063043.1):--------MNTS--LHHGLQQY---RNHSQGAIPVGKPAQ----GERGSASGCYEQLLISTEVFLTLGLVSLLENILVIAAIVKNKNLHSPMYFFICSLAVADLLVSVSNASE :  96
Common carp(XP_018939072.1):-----MNTS--HHHGLQHSY--RNHSQGVLPVGKPAQ----GERGSASGCYEQLLISTEVFLTLGLVSLLENILVIAAIVKNKNLHSPMYFFICSLAVADLLVSVSNASE :  97
Goldfish(XP_026130800.1):--------MNTS--HHHGLHHSY--RNHSQGALPVGKPDQ----GERGSTSGCYEQLLISTEVFLTLGLVSLLENILVIAAIIKNKNLHSPMYFFICSLAVADLLVSVSNASE :  97
Goldfish(XP_026106271.1):--------MNTS--HHHGQHHSY--RNHSQGALPVGKPDQ----GERGSTSGCYEQLLISTEVFLTLGLVSLLENILVIAAIIKNKNLHSPMYFFICSLAVADLLVSVSNASE :  97
Zebrafish(NP_775385.1):----------MNTS--HHHGLHHSF--RNHSQGALPVGKPSH----GDRGSASGCYEQLLISTEVFLTLGLVSLLENILVIAAIVKNKNLHSPMYFFICSLAVADLLVSVSNASE :  97
Stickleback(ENSGACT00000007360):-MNST--QSGGLIQGYHNRSASGVSPP----DNDLAAEGKDASAGCYEQLLISTEVFLTLGIISLLENILVVAAIVKNKNLHSPMYFFICSLAVADMLVSVSNASE :  99
Medaka(XP_004081243.1):----------MNST--LPYGSVPN---RSLSSATLP------PDLGGQKDSSAGCYEQLLISTEVFLTLGIISLLENILVVAAIVKNKNLHSPMYFFICSLAVADMLVSVSNASE :  94
Human(NP_005903.2):-------------MVNST---HRGMHTSLHLWNRSSYRL---HSNASESLGKGYSDGGCYEQLFVSPEVFVTLGVISLLENILVIVAIAKNKNLHSPMYFFICSLAVADMLVSVSNGSE : 100
Cave Fish(ENSAMXT00000027076):---MNIS--QHHGLHHSH--RNHSLVAHAGSKVE-------REPTSGCYEQLLVSTEVFLILGLVSLVENILVIAAIVKNKNFHSPMYFFICSLAAADLLVSLSNSTE :  94
Atlantic cod(XP_030205462.1):----MNAT--QLYGLIQAYHSNASRGAAAPTTQPDVPPDAKEDPSASGCYKQLLISPEVFLTLGIISLLENILVIAAIAKNKNLHSPMYFFICSLAVADMLVSVSNASE : 103
Spotted gar(ENSLOCP00000022262):KMNTT--HHHGLIHNYHSRNFSSGAFTVNNDSNE-----KDSSSGCYEQLLISTEVFLTLGIVSLLENILVIAAIIKNKNLHSPMYFFICSLAVADMLVSVSNAWE :  99
Asian arowana(XP_018596452.1):---MNST--VTQGLTQGHQLRNLSLGGLPPSKDPS----GGRSSSSGCYEQLLISTEVFLTLGIVSLLENILVVAAIVKNKNLHSPMYLFICSLAVADMLVSVSNASE :  99

Arctic char(XP_023855403.1):----TIVIALINGGNLTISGSLIKSMDNVFDSMICSSLLASICSLLAIAIDRYITIFYALRYHNIVTVKRAMAVIACIWSCCVASGVLFIIYSESTTVLICLITMFFTML : 209
Rainbow trout(XP_021476310.1):--TIVIALINGGNLTISGSLIKSMDNVFDSMICSSLLASICSLLAIAIDRYITIFYALRYHNIVTVKRAMAVIACIWSCCVASGVLFIIYSESTTVLICLITMFFTML : 210
Sockeye salmon(XP_029482615.1):-TIVIALINGGNLTISGSLIKSMDNVFDSMICSSLLASICSLLAIAIDRYITIFYALRYHNIVTVKRAMAVIACIWSCCVASGVLFIIYSESTTVLICLITMFFTML : 210
Coho salmon(XP_020364322.1):----TIVIALINGGNLTISGSLIKSMDNVFDSMICSSLLASICSLLAIAIDRYITIFYALRYHNIVTVKRAMAVIACIWSCCVASGVLFIIYSESTTVLICLITMFFTML : 210
Chinook salmon(XP_024230545.1):-TIVIALINSGNLTISGSLIKSMDNVFDSMICSSLLASICSLLAIAIDRYITIFYALRYHNIVTVKRAMAVIACIWSCCVASGVLFIIYSESTTVLICLITMFFTML : 210
Atlantic salmon(XP_014013065.1):TIVIALINGGNLTISGSLIKSMDNVFDSMICSSLLASICSLLAIAIDRYITIFYALRYHNIVTVKRAMAVIVCIWSCCVASGVLFIIYSESTTVLICLITMFFTML : 210
Brown trout(XP_029555723.1):----TIVIALINGGNLTISGSLIKSMDNVFDSMICSSLLASICSLLAIAIDRYITIFYALRYHNIVTVKRAMAVIVCIWSCCVASGVLFIIYSESTTVLICLITMFFTML : 210
Brown trout(XP_029612042.1):----TIVIALINSGNLSISGSLIKSMDNVFDSMICSSLLASICSLLAIAIDRYITIFYALRYHNIVTVKSALAVISLIWSCCVASGVLFIIYSESTTVLICLITMFFSML : 209
Atlantic salmon(XP_014036044.1):TIVIALINSGNLSISGSLIKSMDNVFDSMICSSLLASICSLLAIAIDRYITIFYALRYHNIVTVKSALAVISLIWSCCVASGVLFIIYSESTTVLICLITMFFSML : 209
Chinook salmon(XP_024248133.1):-TIVIALINSGNLSISGSLIKSMDNVFDSMICSSLLASICSLLAIAIDRYITIFYALHYHNIVTVKRALAVISLIWLCCVASGVLFIIYSESTTVLICLITMFFTML : 209
Coho salmon(XP_020349696.1):----TIVIALINSGNLSISGSLIKSMDNVFDSMICSSLLASICSLLAIAIDRYITIFYALHYHNIVTVKRALAVISLIWLCCVASGVLFIIYSESTTVLICLITMFFTML : 209
Sockeye salmon(XP_029518586.1):-TIVIALINSGNLSISGSLIKSMDNVFDSMICSSLLASICSLLAIAIDRYITIFYALHYHNIVTVKRALAVISLIWLCCVASGVLFIIYSESTTVLICLITMFFSML : 209
Arctic char(XP_023991111.1):----TIVIALINSGNLSISGSLIKSMDNVFDSMICSSLLASICSLLAIAIDRYITIFYALRYHNIVTVKRALAVISLIWSCCVASGVLFIIYSESTTVLICLITMFFTML : 209
Rainbow trout(XP_021418765.1):--TIVIALINSGSLSISGSLIKSMDNVFDSMICSSLLASICSLLAIAIDRYITIFYALHYHNIVTVKRALAVISLIWLCCVVSGVLFIIYSESTTVLICLITMFFTML : 209
Northern pike(XP_010903767.1):--TIVIALINGGNLSISGLHIKSMDNVFDSMICSSLLASICSLLAIAIDRYITIFYALRYHNIVTVKRALAVIACIWLCCVASGVLFIIYSESTTVLICLITMFFTML : 207
Chinook salmon(XP_024246605.1):-TIVMAMITDGNLGIGGGMIKSMDNVFDSMICSSLLASIWSLLAIAVDRYVTIFYALRYHNIMTVRRAAAIITSIWTFCTVSGVLFIVYSESTTVLICLIIMFFSML : 208
Sockeye salmon(XP_029476902.1):-TIVMAMITDGNLGIGGGMIKSMDNVFDSMICSSLLASIWSLLAIAVDRYVTIFYALRYHNIMTVRRAAAIITSIWTFCTVSGVLFIVYSESTTVLICLIIMFFSML : 208
Rainbow trout(XP_021445107.1):--TIVMAMITDGNLGIGGGMIKSMDNVFDSMICSSLLASIWSLLAIAVDRYVTIFYALRYHNIMTVRRAATIITSIWTFCTVSGVLFIVYSESTTVLICLIIMFFSML : 208
Coho salmon(XP_020318023.1):----TIVMAMITDGNLGIGGGMIKSMDNVFDSMICSSLLASIWSLLAIAVDRYVTIFYALRYHNIMTVRRAASIITSIWTFCTVSGVLFIVYSESTTVLICLIIMFFSML : 208
Arctic char(XP_023865987.1):----TIVMAMITDGNLGIGGGMIKSMDNVFDSMICSSLLASIWSLLAIAVDRYVTIFYALRYHNIMTVRRAAAIITSIWTFCTVSGVLFIVYSESTTVLICLIIMFFSML : 208
Brown trout(XP_029581515.1):----TIVMAMITDGNLGIGGGKIKSMDNVFDSMICSSLLASIWNLLAIAVDRYVTIFYALRYHNIMTVRRAAAIITSIWTFCTVSGVLFIVYSESTTVLICLIIMFFSML : 208
Atlantic salmon(XP_014045837.1):TIVMAMITDGNLGIGGGKIKSMDNVFDSMICSSLLASIWNLLAIAVDRYVTIFYALRYHNIMTVRRAAAIITSIWTFCTVSGVLFIVYSESTTVLICLIIMFFSML : 208
Brown trout(XP_029562072.1):----TIVMATITDGNLGIGGGVIKSMDNVFDSMICSSLLASIWSLLAIAVDRYVTIFYALRYHNIMTTRRAAAIITSIWTLCTVSGVLFIVYSESTTVLICLIIMFFSML : 208
Atlantic salmon(XP_013995955.1):TIVMATITDGNLGIGGGVIKSMDNVFDSMICSSLLASIWSLLAIAVDRYVTIFYALRYHNIMTTRRAAAIITSIWTLCTVSGVLFIVYSESTTVLICLIIMFFSML : 208
Rainbow trout(XP_021468992.1):--TIVMAMITDGNLGIGGGVIKSMDNVFDSMICSSLLASIWSLLAIAVDRYVTIFYALRYHNIMTTRRAAAIITSIWTFCTVSGVLFIVYSESTTVLICLIIMFFSML : 208
Coho salmon(XP_020322312.1):----TIVMAMITDGNLGIGGGVIKSMDNVFDSMICSSLLASIWSLLAIAVDRYVTIFYALRYHNIMTTRRAAAIITSIWTFCTVSGILFIVYSESTTVLICLIIMFFSML : 208
Sockeye salmon(XP_029497744.1):-TIVMAMITDGNLGIGGGVIKSMDNVFDSMICSSLLASIWSLLAIAVDRYVTIFYALRYHNIMTTRRAAAIITSIWTFCTVSGVLFIVYSESTTVLICLIIMFFSML : 208
Chinook salmon(XP_024292220.1):-TIVMAMITDGNLGIGGGVIKSMDNVFDSMICSSLLASIWSLLAIAVDRYVTIFYALRYHNIMTTRRAAAIITSIWTFCTVSGVLFIVYSESTTVLICLIIMFFSML : 208
Arctic char(XP_023833474.1):----TIVMAMITDGNLGIGGGVIKSMDNVFDSMICSSLLASIWSLLAIAVDRYVTIFYALRYHNIMTTRRAAAIITSIWTFCTVSGVLFIVYSESTTVLICLIIMFFSML : 208
Northern pike(XP_010889688.1):--TVVMVWITDSNIEMGGGVVKSVDNVFDSMICSSLLASIWSLLAIAVDRYVTIFYALRYHNIMTVHRAAAIITSIWTFCTVSGVLFIIYSESTTVLICLIVMFFSML : 208
Goldfish(XP_026063043.1):-------TVVMALITGGNLTNRESIIKNMDNIFDSMICSSLLASIWSLLAIAVDRYITIFYALRYHNIMTQRRAGTIITCIWTFCMVSGVLFIVYSESTTVLICLISMFFTML : 202
Common carp(XP_018939072.1):----TVVMALITGGNLTNRESIIKNMDNIFDSMICSSLLASIWSLLAIAVDRYITIFYALRYHNIMTQRRAGTIITCIWTFCMVSGVLFIVYSESTTVLICLISMFFTML : 203
Goldfish(XP_026130800.1):-------TVVMALITGGNLTYRESIIKNMDNIFDSMICSSLLASIWSLLAIAVDRYITIFYALRYHNIMTQRRAGTIITCIWTLCTVSGVLFIVYSESTTVLICLISMFFTML : 203
Goldfish(XP_026106271.1):-------TVVMALITGGNLTYRESIIKNMDNIFDSMICSSLLASIWSLLAIAVDRYITIFYALRYHNIMTQRRAGTIITCIWTLCTVSGVLFIVYSESTTVLICLISMFFTML : 203
Zebrafish(NP_775385.1):---------TVVMALITGGNLTNRESIIKNMDNVFDSMICSSLLASIWSLLAIAVDRYITIFYALRYHNIMTQRRAGTIITCIWTFCTVSGVLFIVYSESTTVLICLISMFFTML : 203
Stickleback(ENSGACT00000007360):TIVIALIAGGTLTIPVALIRNMDNVFDSMICSSLLASICSLLAIAVDRYITIFYALRYHNIVTLRRATLVIGSIWTCCTVSGILFIIYSESTSVLICLITMFFTML : 205
Medaka(XP_004081243.1):---------TIVIALINGGNLSIPVRLIKSMDNVFDSMICSSLLASICSLLAIAVDRYITIFYALRYHNIVTLRRAAVVISSIWTCCIVSGILFIIYSESTTVLICLITMFFTML : 200
Human(NP_005903.2):-------------TIVITLLNSTDTD-AQSFTVNIDNVIDSVICSSLLASICSLLSIAVDRYFTIFYALQYHNIMTVKRVGIIISCIWAACTVSGILFIIYSDSSAVIICLITMFFTML : 205
Cave Fish(ENSAMXT00000027076):--TVVMALITGGNLSISGGVAKTMDNVFDSMICSSLLASIWNLLAIAIDRYITIFYGLRYHNIMTQKRAAIVIVCIWAFCTASGVLFIVYSESPTVLICLISMFFAML : 200
Atlantic cod(XP_030205462.1):---TIVISLINSGSLPIPVTLIKSMDNVFDSLICSSLLASICSLLAIAVDRYITIFYALRYHNIVTVRRAMLVIAGIWACCTGSGVLFIVYSESTTVLICLITMFLTML : 209
Spotted gar(ENSLOCP00000022262):TIVMALITSGHLTIQDNLIKNMDNVFDSMICSSLLASICSLLAIAIDRYITIFYALRYHNIMTMRRAMMIITTIWAFCTISGILFIIYSESTTVIICLITMFFTML : 205
Asian arowana(XP_018596452.1):--TIVMALITGSHLSIGASLIKSMDNVFDSMICSSLLASICSLLAIAVDRYITIFYALRYHNIMTVRRALTIISCIWTFCTVSGILFIIYSESTTVLICLITMFFTML : 205
```

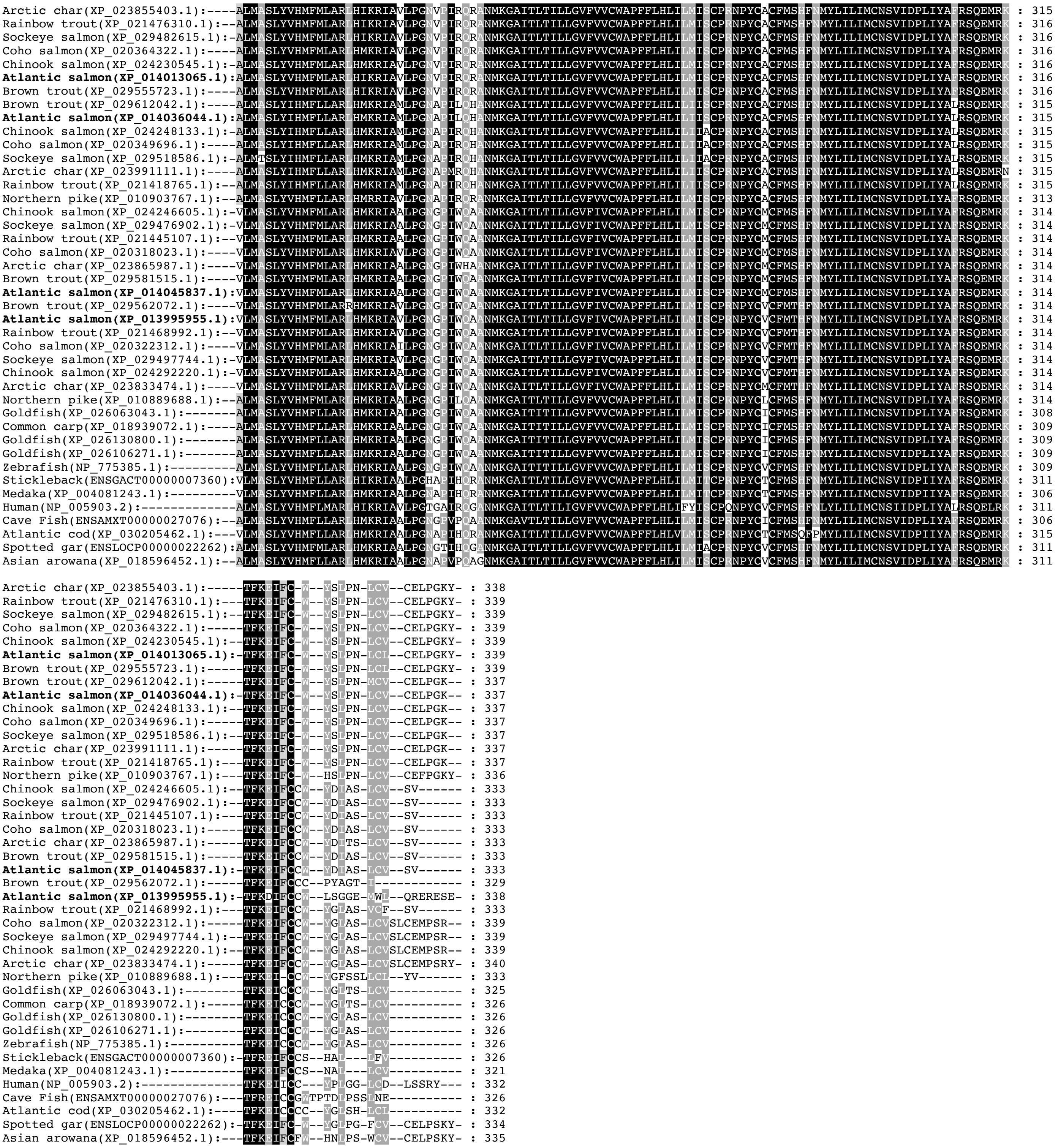

Supplementary Figure 2. Multiple sequence alignment of Mc4r. The Atlantic salmon MC4R predicted amino acid sequences were aligned with those of other vertebrate species by using the Multiple Sequence Alignment in MUSCLE. Conserved amino acids among the species are highlighted in black. Protein ID accession numbers are shown after the species name.

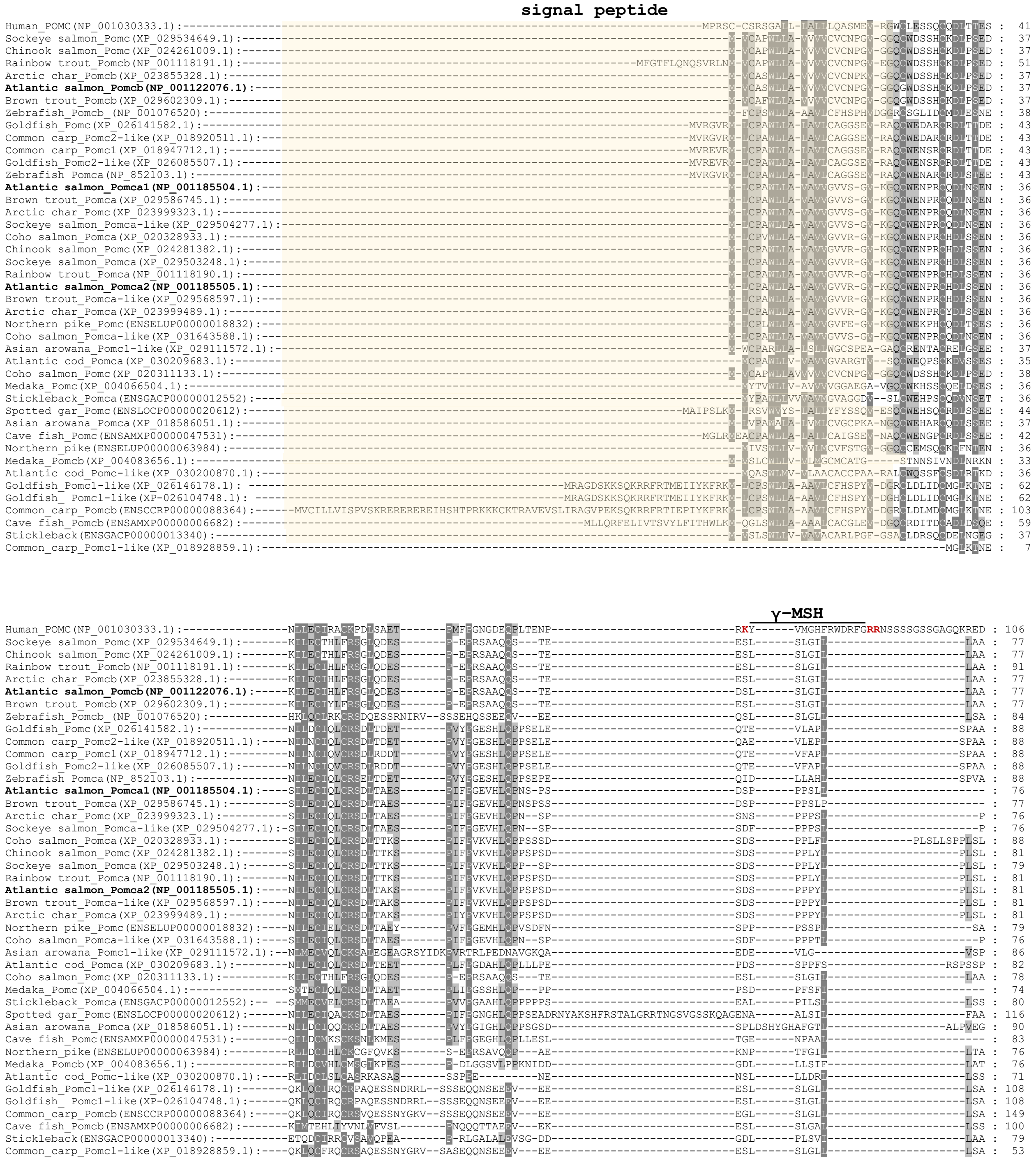
signal peptide
γ-MSH

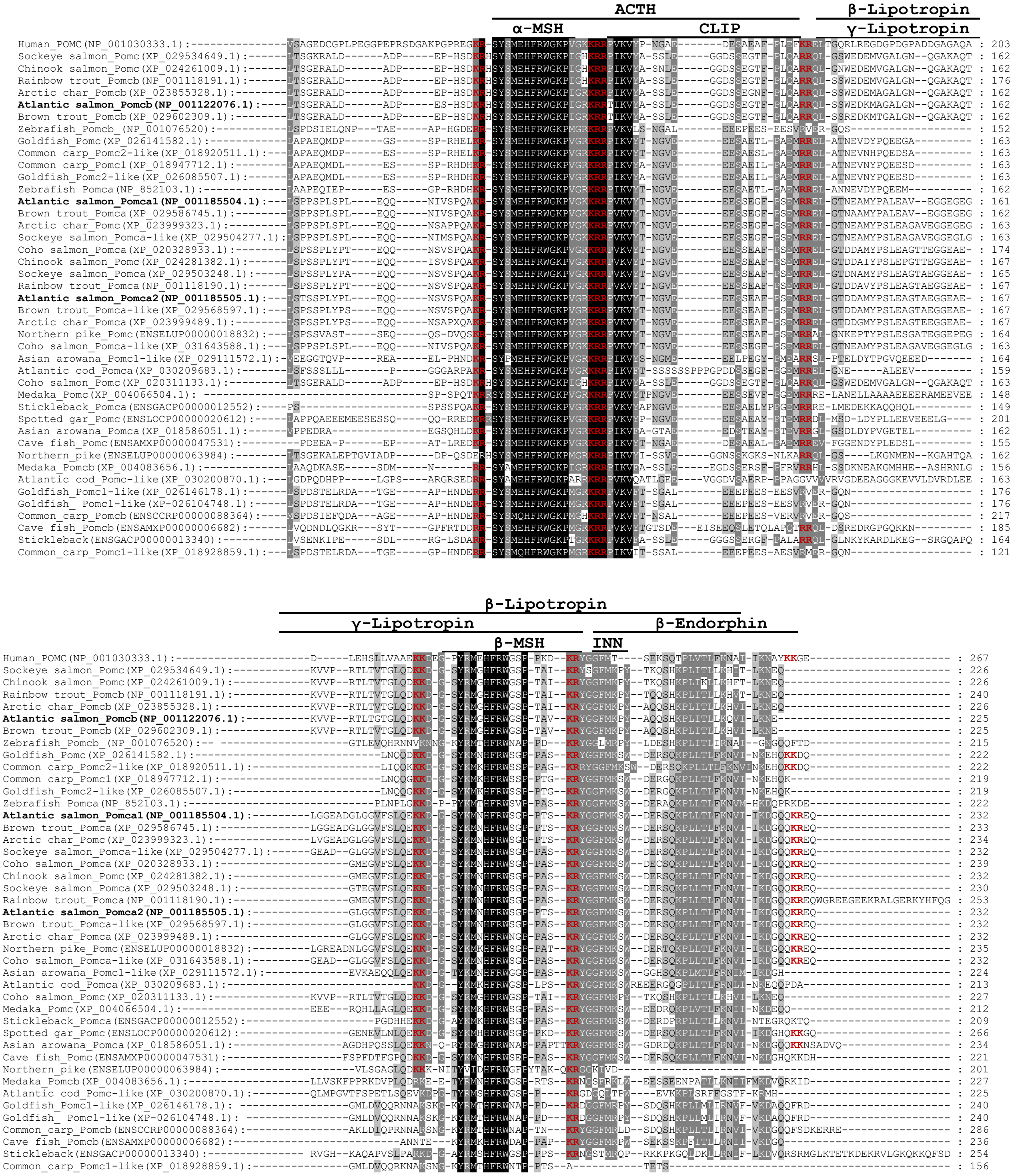

Supplementary Figure 3. Multiple sequence alignment of Pomc. The Atlantic salmon deduced amino acid sequences were aligned with other vertebrate homologues by using the Multiple Sequence Alignment tool in MUSCLE. Conserved amino acids among the species are highlighted in black. Potential cleavage sites of Pomc precursor are bold-marked in red and potential cleaved peptide names are shown on top of the alignment. Protein ID accession numbers are shown after the species name.

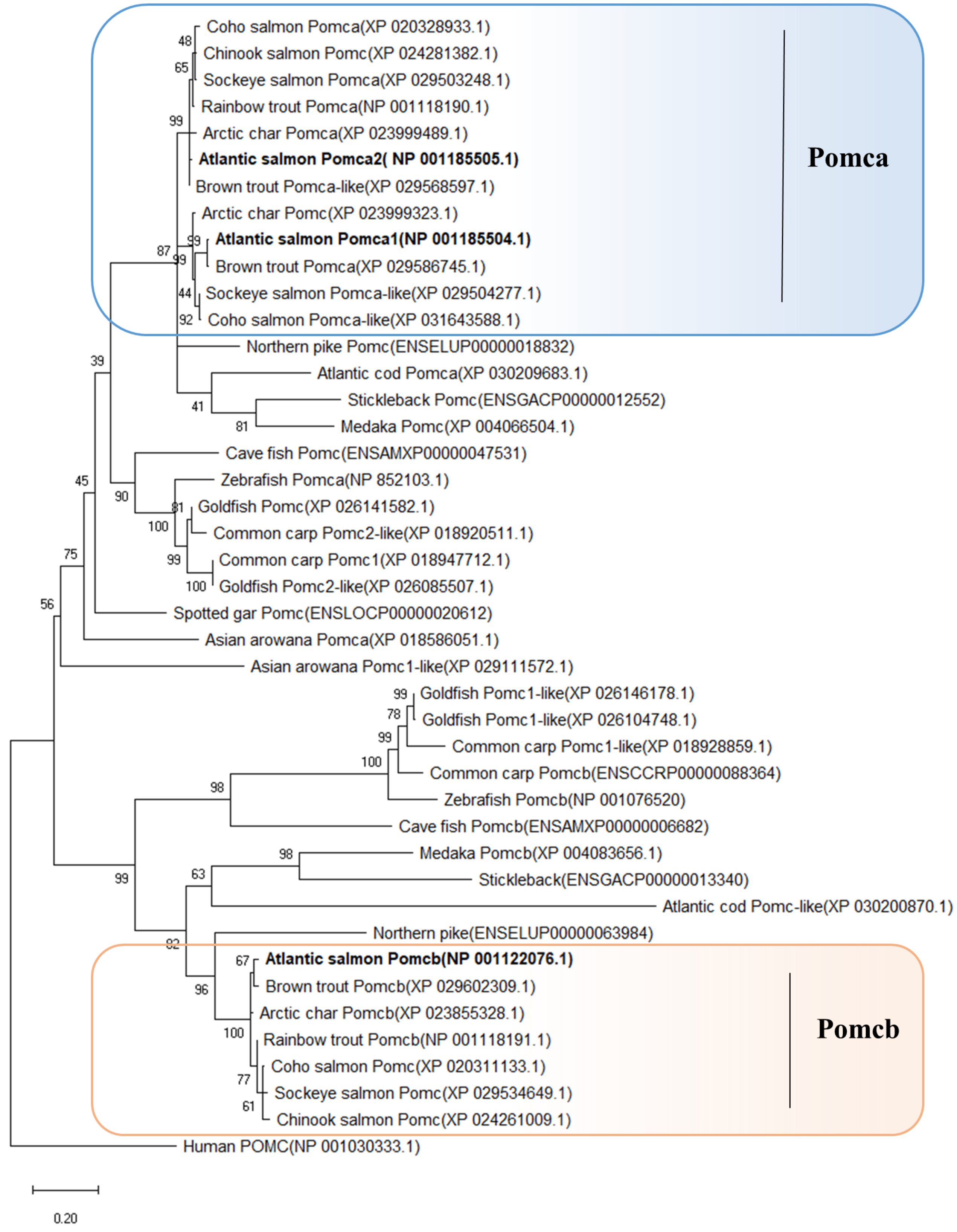

Supplementary Figure 4. Phylogenetic relationship of Pomc in Salmonidae family. The phylogenetic tree was constructed using the predicted mature peptide sequences with the Maximum Likelihood method (1000 bootstraps replicates) and JTT+G+I matrix-based model in MEGA X. The tree with the highest log likelihood (-3209.08) is shown. The percentage of trees in which the associated taxa clustered together is shown next to the branches. Phylogenic tree was rooted to the human Pomc sequence.

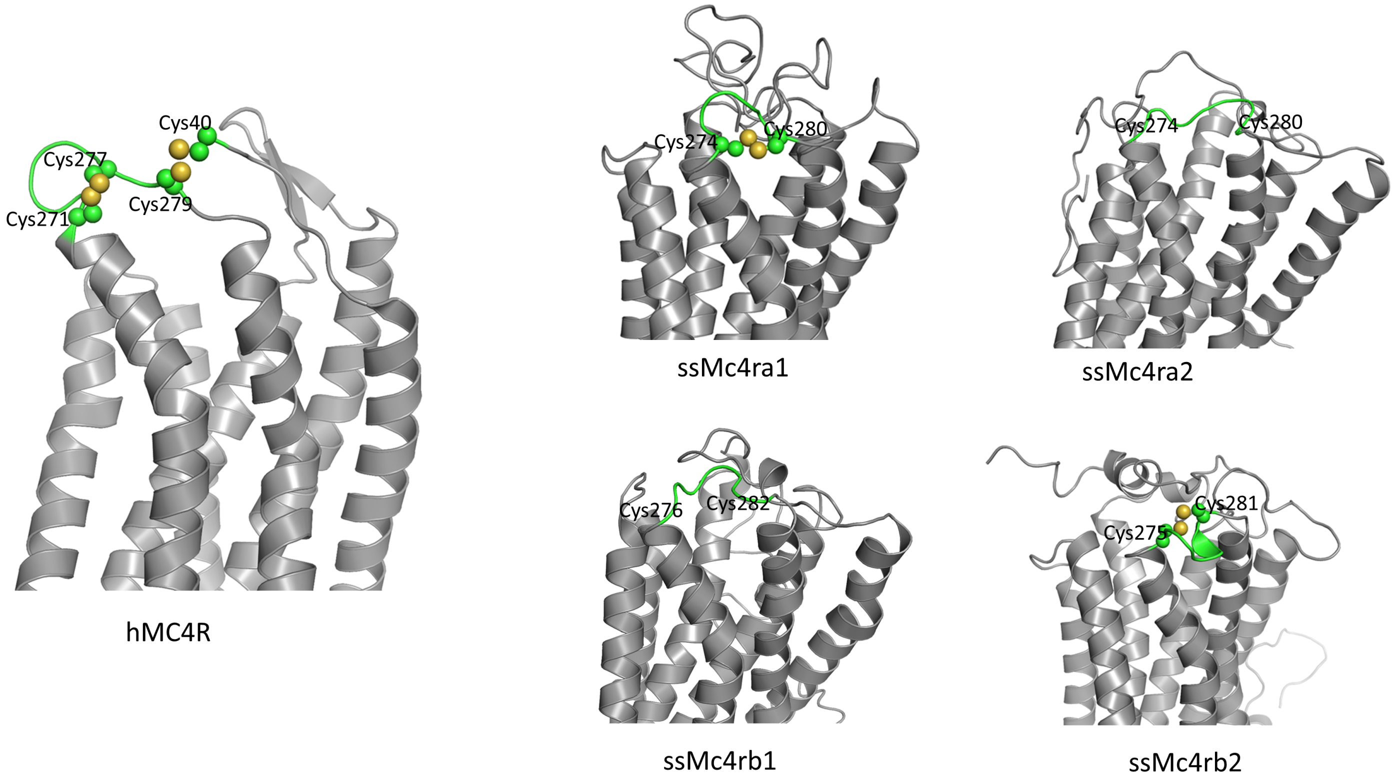

Supplementary Figure 5. Overview of the disulfide bonds in human and Atlantic salmon MC4R. Tertiary protein structures and disulfide bonds of human MC4R (UniProt) and Atlantic salmon Mc4r (IntFOLD) edited in PyMOL Molecular Graphics System v 2.3.

Supplementary Table 1. Statistical test and *p*-values for the Condition factor (*K*)

Test Details	Fed-Before	Fed-After	Fasted-Before	Fasted-After
Number of values	21	27	21	26
Minimum	0.9836	0.8749	1.063	0.8914
Maximum	1.316	1.248	1.224	1.149
Range	0.3322	0.3726	0.1607	0.2579
Mean	1.129	1.095	1.162	1.035
Std. Deviation	0.0744	0.08044	0.04434	0.05556
Std. Error of Mean	0.01624	0.01548	0.009675	0.0109
**Sidak's multiple comparisons test**				
Mean Diff,		-0.03247		0.05996
95,00% CI of diff,		-0.07873 to 0.01379		0.01877 to 0.1011
P Value		0.2149		**0.0027

Supplementary Table 2. Statistical test and p-values for each gene expression per brain region between treatments.

Brain Region	*pomca1*		*pomca2*		*pomcb*		*agrp1*		*agrp2*		*mc4ra1*		*mc4ra2*		*mc4rb1*		*mc4rb2*	
	**Test**	***p*- value**	**Test**	***p*- value**	**Test**	***p*- value**	**Test**	***p*- value**	**Test**	***p*- value**	**Test**	***p*- value**	**Test**	***p*- value**	**Test**	***p*- value**	**Test**	***p*- value**
OB	t-test	**0.0057	t-test	0.1019	t-test	0.2286	t-test	0.6273	t-test	0.3691	t-test	0.3053	t-test	0.3236	Mann Whitney test	0.3290	t-test	0.3421
TEL	t-test	0.0873	t-test	* 0.0233	Mann Whitney test	*0.0303	t-test	0.7904	t-test	0.3404	t-test	0.4419	t-test	0.3660	t-test	0.2858	t-test	0.1719
**MB**	t-test	0.6014	t-test	0.7722	Mann Whitney test	0.4286	t-test	0.5160	t-test	0.1885	t-test	0.5204	t-test	0.3374	t-test	0.3284	t-test	0.7966
**CE**	t-test	0.1819	t-test	*0.0340	t-test	0.2020	t-test	0.9507	Mann Whitney test	0.5368	Mann Whitney test	0.4286	t-test	0.6154	t-test	0.4260	t-test	0.5275
**HYP**	t-test	0.4448	t-test	0.1073	Mann Whitney test	0.2468	Mann Whitney test	0.4286	t-test	0.4249	t-test	0.8997	t-test	0.5308	t-test	0.5465	t-test	0.7020
**SV**	t-test	0.2200	Mann Whitney test	0.1175	t-test	0.6891	Mann Whitney test	0.5368	Mann Whitney test	0.5368	Mann Whitney test	0.1255	Mann Whitney test	0.5022	t-test	0.1680	Mann Whitney test	>0.9999
**PT**	t-test	0.1775	t-test	0.983	Mann Whitney test	0.7922	t-test	0.0895	t-test	0.2765	t-test	0.1502	t-test	0.5521	t-test	0.4346	t-test	0.9436
**BS**	t-test	0.1710	t-test	0.930	t-test	0.1585	t-test	0.3394	t-test	0.1684	t-test	0.3213	t-test	0.2160	t-test	0.2398	t-test	0.5583

Supplementary Table 3. Protein sequence identity matrix of human and Actinopterigii Mc4r

	Arctic_char_XP_023855403.1_	Rainbow_trout_XP_021476310.1_	Sockeye_salmon_XP_029482615.1_	Coho_salmon_XP_020364322.1_	Chinook_salmon_XP_024230545.1_	Atlantic_salmon_XP_014013065.1	Brown_trout_XP_029555723.1_	Brown_trout_XP_029612042.1__	Atlantic_salmon_XP_014036044.1	Chinook_salmon_XP_024248133.1_	Coho_salmon_XP_020349696.1_	Sockeye_salmon_XP_029518586.1_	Arctic_char_XP_023991111.1_	Rainbow_trout_XP_021418765.1_	Northern_pikeXP_010903767.1	Chinook_salmon_XP_024246605.1_	Sockeye_salmon_XP_029476902.1_	Rainbow_trout_XP_021445107.1_	Coho_salmon_XP_020318023.1_	Arctic_char_XP_023865987.1_	Brown_trout_XP_029581515.1_	Atlantic_salmon_XP_014045837.1	Brown_trout_XP_029562072.1_	Atlantic_salmon_XP_013995955.1	Rainbow_trout_XP_021468992.1_	Coho_salmon_XP_020322312.1_	Sockeye_salmon_XP_029497744.1_	Chinook_salmon_XP_024292220.1_	Arctic_char_XP_023833474.1_	Northern_pikeXP_010889688.1	Goldfish_XP_026063043.1_	Common_carp_XP_018939072.1_	Goldfish_XP_026130800.1_	Goldfish_XP_026106271.1_	Zebrafish_NP_775385.1_	Stickleback_ENSGACT00000007360	Medaka_XP_004081243.1	Human_NP_005903.2_	Cave_Fish_ENSAMXT00000027076	Atlantic_cod_XP_030205462.1	Spotted_gar_ENSLOCP0000002226	Asian_arowana__XP_018596452.1
Arctic_char_XP_023855403.1_	338	99%	99%	99%	99%	98%	98%	90%	90%	90%	90%	89%	91%	90%	90%	76%	76%	75%	76%	75%	75%	75%	74%	74%	75%	76%	76%	76%	76%	74%	76%	76%	76%	76%	76%	77%	79%	68%	72%	76%	81%	81%
	0	99%	99%	99%	99%	99%	98%	95%	95%	96%	96%	95%	96%	95%	94%	82%	82%	82%	83%	82%	82%	82%	81%	82%	83%	84%	84%	84%	84%	81%	84%	84%	84%	84%	84%	84%	86%	80%	82%	83%	88%	88%
	0	0%	0%	0%	0%	0%	0%	0%	0%	0%	0%	0%	0%	0%	1%	3%	3%	3%	3%	3%	3%	3%	4%	1%	3%	2%	2%	2%	2%	3%	4%	4%	4%	4%	4%	4%	5%	2%	5%	2%	1%	1%
Rainbow_trout_XP_021476310.1_	338	339	99%	99%	99%	98%	98%	89%	89%	90%	90%	88%	90%	89%	91%	76%	76%	75%	76%	75%	75%	75%	74%	74%	75%	75%	76%	76%	76%	74%	76%	76%	76%	76%	76%	77%	79%	69%	72%	76%	81%	81%
	338	0	99%	100%	99%	99%	99%	95%	95%	96%	96%	95%	96%	95%	94%	82%	82%	82%	83%	82%	82%	82%	81%	82%	83%	84%	84%	84%	84%	81%	84%	84%	84%	83%	84%	84%	85%	81%	82%	83%	88%	88%
	1	0	0%	0%	0%	0%	0%	0%	0%	0%	0%	0%	0%	0%	0%	3%	3%	3%	3%	3%	3%	3%	4%	2%	3%	2%	2%	2%	2%	4%	4%	4%	4%	4%	4%	4%	5%	2%	6%	2%	1%	1%
Sockeye_salmon_XP_029482615.1_	337	338	339	99%	99%	98%	98%	89%	89%	90%	90%	88%	90%	89%	91%	76%	76%	75%	76%	75%	75%	75%	74%	74%	75%	75%	76%	76%	76%	74%	76%	76%	76%	76%	76%	77%	79%	69%	72%	76%	81%	81%
	337	338	0	99%	99%	99%	98%	95%	95%	96%	96%	95%	96%	95%	94%	82%	82%	82%	83%	82%	82%	82%	81%	82%	83%	84%	84%	84%	84%	81%	84%	84%	84%	83%	84%	84%	85%	81%	82%	83%	88%	88%
	1	0	0	0%	0%	0%	0%	0%	0%	0%	0%	0%	0%	0%	0%	3%	3%	3%	3%	3%	3%	3%	4%	2%	3%	2%	2%	2%	2%	4%	4%	4%	4%	4%	4%	4%	5%	2%	6%	2%	1%	1%
Coho_salmon_XP_020364322.1_	336	337	336	339	99%	98%	97%	90%	90%	90%	90%	89%	91%	90%	91%	76%	76%	76%	76%	75%	76%	75%	74%	74%	75%	76%	76%	76%	77%	74%	76%	76%	77%	76%	77%	77%	79%	68%	72%	76%	81%	81%
	338	339	338	0	99%	99%	99%	95%	95%	96%	96%	95%	96%	95%	94%	82%	82%	82%	83%	82%	82%	82%	81%	82%	83%	84%	84%	84%	84%	81%	84%	84%	84%	83%	84%	84%	85%	81%	82%	83%	88%	88%
	1	0	0	0	0%	0%	0%	0%	0%	0%	0%	0%	0%	0%	0%	3%	3%	3%	3%	3%	3%	3%	4%	2%	3%	2%	2%	2%	2%	4%	4%	4%	4%	4%	4%	4%	5%	2%	6%	2%	1%	1%
Chinook_salmon_XP_024230545.1_	336	337	336	337	339	98%	97%	90%	90%	90%	90%	89%	91%	90%	91%	76%	76%	76%	76%	75%	76%	75%	74%	74%	75%	76%	76%	76%	77%	74%	76%	76%	76%	76%	76%	76%	79%	69%	72%	77%	82%	81%
	337	338	337	338	0	99%	98%	95%	95%	96%	96%	95%	96%	95%	94%	82%	82%	82%	83%	82%	82%	82%	81%	82%	83%	84%	84%	84%	84%	81%	84%	84%	83%	83%	83%	84%	85%	81%	81%	83%	88%	87%
	1	0	0	0	0	0%	0%	0%	0%	0%	0%	0%	0%	0%	0%	3%	3%	3%	3%	3%	3%	3%	4%	2%	3%	2%	2%	2%	2%	4%	4%	4%	4%	4%	4%	4%	5%	2%	6%	2%	1%	1%
Atlantic_salmon_XP_014013065.1	334	335	334	333	333	339	99%	89%	89%	89%	89%	88%	90%	89%	90%	76%	76%	75%	76%	75%	75%	75%	74%	75%	75%	75%	76%	76%	76%	74%	76%	76%	76%	76%	76%	76%	79%	68%	72%	76%	81%	80%
	336	337	336	337	336	0	99%	94%	94%	95%	95%	94%	95%	94%	94%	82%	82%	82%	82%	82%	81%	81%	80%	81%	83%	84%	84%	83%	84%	80%	84%	84%	83%	83%	83%	84%	85%	80%	82%	83%	88%	87%
	1	0	0	0	0	0	0%	0%	0%	0%	0%	0%	0%	0%	0%	3%	3%	3%	3%	3%	3%	3%	4%	2%	3%	2%	2%	2%	2%	4%	4%	4%	4%	4%	4%	4%	5%	2%	6%	2%	1%	1%
Brown_trout_XP_029555723.1_	333	334	333	332	332	338	339	89%	89%	89%	89%	87%	90%	89%	89%	76%	76%	75%	76%	75%	75%	75%	74%	75%	75%	75%	76%	76%	76%	74%	76%	76%	76%	76%	76%	76%	79%	68%	72%	77%	81%	80%
	335	336	335	336	335	338	0	94%	94%	95%	95%	94%	95%	94%	94%	82%	82%	81%	82%	81%	81%	81%	80%	81%	82%	83%	83%	83%	84%	80%	84%	84%	84%	83%	84%	84%	85%	81%	82%	83%	88%	87%
	1	0	0	0	0	0	0	0%	0%	0%	0%	0%	0%	0%	0%	3%	3%	3%	3%	3%	3%	3%	4%	2%	3%	2%	2%	2%	2%	4%	4%	4%	4%	4%	4%	4%	5%	2%	6%	2%	1%	1%
Brown_trout_XP_029612042.1__	305	305	305	306	307	304	303	337	98%	97%	97%	96%	97%	96%	89%	75%	75%	75%	75%	75%	75%	75%	73%	74%	75%	75%	76%	76%	75%	73%	75%	75%	75%	75%	75%	75%	78%	67%	71%	76%	79%	79%
	324	324	324	324	325	322	321	0	99%	98%	98%	97%	98%	97%	93%	82%	82%	82%	83%	82%	82%	82%	81%	82%	83%	85%	85%	84%	84%	82%	84%	84%	83%	83%	83%	84%	85%	80%	81%	84%	87%	86%
	1	2	2	2	2	2	2	0	0%	0%	0%	0%	0%	0%	1%	2%	2%	2%	2%	2%	2%	2%	4%	2%	2%	2%	2%	2%	2%	3%	4%	3%	3%	3%	3%	3%	5%	2%	5%	2%	2%	1%
Atlantic_salmon_XP_014036044.1	305	305	305	306	307	303	302	333	337	96%	96%	95%	97%	95%	90%	75%	75%	75%	75%	75%	75%	75%	73%	73%	75%	75%	76%	76%	75%	74%	76%	76%	76%	76%	76%	75%	78%	67%	71%	75%	80%	79%
	324	324	324	323	325	321	320	334	0	97%	97%	97%	97%	96%	94%	82%	82%	82%	82%	82%	82%	82%	81%	82%	83%	84%	84%	84%	84%	82%	84%	84%	84%	83%	84%	84%	85%	80%	81%	84%	88%	86%
	1	2	2	2	2	2	2	0	0	0%	0%	0%	0%	0%	1%	2%	2%	2%	2%	2%	2%	2%	4%	2%	2%	2%	2%	2%	2%	3%	4%	3%	3%	3%	3%	3%	5%	2%	5%	2%	2%	1%
Chinook_salmon_XP_024248133.1_	306	306	306	307	308	305	304	327	325	337	99%	97%	97%	97%	90%	75%	75%	75%	75%	75%	75%	75%	73%	73%	75%	75%	76%	76%	75%	73%	75%	75%	76%	75%	76%	75%	78%	68%	71%	76%	80%	79%
	326	326	326	326	327	324	323	332	329	0	100%	99%	99%	99%	94%	82%	82%	82%	83%	82%	82%	82%	82%	82%	83%	85%	85%	84%	84%	82%	83%	83%	83%	83%	83%	84%	84%	80%	81%	84%	87%	86%
	1	2	2	2	2	2	2	0	0	0	0%	0%	0%	0%	1%	2%	2%	2%	2%	2%	2%	2%	4%	2%	2%	2%	2%	2%	2%	3%	4%	3%	3%	3%	3%	3%	5%	2%	5%	2%	2%	1%
Coho_salmon_XP_020349696.1_	306	306	306	307	308	305	304	328	326	336	337	98%	98%	97%	90%	75%	75%	75%	75%	75%	75%	75%	73%	73%	75%	75%	76%	76%	75%	73%	75%	75%	76%	75%	76%	75%	78%	68%	71%	76%	80%	79%
	326	326	326	326	327	324	323	332	329	337	0	99%	99%	99%	94%	82%	82%	82%	83%	82%	82%	82%	82%	82%	83%	85%	85%	84%	84%	82%	83%	83%	83%	83%	83%	84%	84%	80%	81%	84%	87%	86%
	1	2	2	2	2	2	2	0	0	0	0	0%	0%	0%	1%	2%	2%	2%	2%	2%	2%	2%	4%	2%	2%	2%	2%	2%	2%	3%	4%	3%	3%	3%	3%	3%	5%	2%	5%	2%	2%	1%
Sockeye_salmon_XP_029518586.1_	301	301	301	302	303	299	298	324	323	330	331	337	96%	96%	89%	75%	75%	75%	75%	74%	75%	75%	73%	73%	74%	75%	75%	75%	75%	72%	74%	74%	75%	74%	75%	74%	78%	67%	71%	75%	79%	79%
	323	323	323	323	324	321	320	329	327	334	334	0	98%	98%	94%	82%	82%	81%	82%	81%	82%	82%	81%	82%	83%	84%	84%	84%	84%	81%	83%	83%	82%	82%	82%	83%	84%	79%	81%	84%	87%	85%
	1	2	2	2	2	2	2	0	0	0	0	0	0%	0%	1%	2%	2%	2%	2%	2%	2%	2%	4%	2%	2%	2%	2%	2%	2%	3%	4%	3%	3%	3%	3%	3%	5%	2%	5%	2%	2%	1%
Arctic_char_XP_023991111.1_	308	308	308	309	310	307	306	329	327	330	331	325	337	97%	90%	75%	75%	75%	75%	75%	75%	75%	73%	73%	75%	75%	76%	76%	75%	73%	75%	75%	76%	75%	76%	75%	78%	68%	72%	76%	79%	80%
	327	327	327	327	328	325	324	332	329	334	334	331	0	98%	94%	83%	83%	82%	83%	82%	82%	82%	82%	82%	84%	85%	85%	85%	85%	82%	84%	84%	83%	83%	83%	84%	85%	80%	81%	84%	88%	87%
	1	2	2	2	2	2	2	0	0	0	0	0	0	0%	1%	2%	2%	2%	2%	2%	2%	2%	4%	2%	2%	2%	2%	2%	2%	3%	4%	3%	3%	3%	3%	3%	5%	2%	5%	2%	2%	1%
Rainbow_trout_XP_021418765.1_	305	305	305	306	307	304	303	324	322	329	330	324	328	337	90%	75%	75%	75%	75%	75%	75%	75%	73%	73%	75%	75%	76%	76%	75%	73%	75%	75%	75%	75%	75%	75%	79%	68%	70%	76%	79%	80%
	324	324	324	324	325	322	321	329	326	335	335	332	332	0	93%	83%	83%	83%	83%	83%	82%	82%	82%	83%	84%	85%	85%	85%	85%	82%	83%	83%	83%	83%	83%	84%	85%	80%	80%	84%	87%	86%
	1	2	2	2	2	2	2	0	0	0	0	0	0	0	1%	2%	2%	2%	2%	2%	2%	2%	4%	2%	2%	2%	2%	2%	2%	3%	4%	3%	3%	3%	3%	3%	5%	2%	5%	2%	2%	1%
Northern_pikeXP_010903767.1	308	309	309	309	309	306	305	305	307	307	308	305	308	306	336	75%	75%	75%	75%	74%	75%	75%	73%	74%	74%	75%	75%	75%	76%	72%	76%	76%	76%	76%	76%	76%	79%	67%	72%	75%	79%	80%
	321	322	322	322	321	320	319	317	319	320	320	320	319	318	0	80%	80%	80%	81%	80%	81%	81%	79%	80%	81%	82%	82%	82%	82%	80%	83%	83%	82%	82%	82%	82%	84%	78%	81%	83%	87%	86%
	4	3	3	3	3	3	3	5	5	5	5	5	5	5	0	4%	4%	4%	4%	4%	4%	4%	5%	2%	4%	3%	3%	3%	3%	4%	3%	3%	3%	3%	3%	5%	6%	3%	5%	3%	0%	0%
Chinook_salmon_XP_024246605.1_	260	260	260	261	261	260	260	258	258	258	258	256	258	258	258	333	100%	99%	99%	98%	98%	98%	90%	89%	93%	92%	92%	92%	92%	87%	81%	81%	82%	82%	82%	75%	78%	65%	75%	74%	78%	78%
	283	283	283	283	283	282	281	282	281	282	282	279	283	284	277	0	100%	99%	99%	99%	99%	99%	93%	92%	96%	94%	94%	94%	94%	92%	87%	86%	87%	87%	87%	84%	85%	77%	83%	82%	84%	84%
	11	12	12	12	12	12	12	10	10	10	10	10	10	10	15	0	0%	0%	0%	0%	0%	0%	1%	1%	0%	1%	1%	1%	2%	0%	2%	2%	2%	2%	2%	3%	3%	4%	4%	2%	4%	4%
Sockeye_salmon_XP_029476902.1_	260	260	260	261	261	260	260	258	258	258	258	256	258	258	258	333	333	99%	99%	98%	98%	98%	90%	89%	93%	92%	92%	92%	92%	87%	81%	81%	82%	82%	82%	75%	78%	65%	75%	74%	78%	78%
	283	283	283	283	283	282	281	282	281	282	282	279	283	284	277	333	0	99%	99%	99%	99%	99%	93%	92%	96%	94%	94%	94%	94%	92%	87%	86%	87%	87%	87%	84%	85%	77%	83%	82%	84%	84%
	11	12	12	12	12	12	12	10	10	10	10	10	10	10	15	0	0	0%	0%	0%	0%	0%	1%	1%	0%	1%	1%	1%	2%	0%	2%	2%	2%	2%	2%	3%	3%	4%	4%	2%	4%	4%
Rainbow_trout_XP_021445107.1_	259	259	259	260	260	259	259	257	257	257	257	255	257	257	257	332	332	333	99%	98%	98%	98%	90%	89%	93%	91%	92%	92%	91%	86%	81%	82%	82%	82%	83%	75%	78%	65%	75%	74%	78%	78%
	282	282	282	282	282	281	280	281	280	281	281	278	282	283	276	332	332	0	99%	98%	98%	98%	93%	92%	96%	94%	94%	94%	93%	91%	87%	87%	88%	88%	88%	84%	85%	77%	83%	82%	84%	84%
	11	12	12	12	12	12	12	10	10	10	10	10	10	10	15	0	0	0	0%	0%	0%	0%	1%	1%	0%	1%	1%	1%	2%	0%	2%	2%	2%	2%	2%	3%	3%	4%	4%	2%	4%	4%
Coho_salmon_XP_020318023.1_	260	260	260	261	261	260	260	257	257	257	257	255	257	257	258	331	331	331	333	98%	98%	97%	90%	89%	93%	91%	92%	91%	92%	86%	81%	81%	82%	82%	82%	74%	78%	65%	75%	74%	78%	77%
	284	284	284	284	284	283	282	283	282	283	283	280	284	285	278	332	332	332	0	98%	98%	98%	93%	93%	96%	94%	94%	94%	94%	91%	87%	86%	87%	87%	87%	83%	85%	77%	83%	82%	84%	84%
	11	12	12	12	12	12	12	10	10	10	10	10	10	10	15	0	0	0	0	0%	0%	0%	1%	1%	0%	1%	1%	1%	2%	0%	2%	2%	2%	2%	2%	3%	3%	4%	4%	2%	4%	4%
Arctic_char_XP_023865987.1_	258	258	258	259	259	258	258	256	256	256	256	254	256	256	256	329	329	328	327	333	98%	97%	90%	89%	93%	91%	91%	91%	91%	86%	81%	81%	81%	81%	82%	74%	77%	64%	75%	74%	78%	77%
	282	282	282	282	282	281	280	281	280	281	281	278	282	283	276	330	330	329	329	0	98%	98%	93%	92%	95%	93%	93%	94%	93%	91%	87%	86%	87%	87%	87%	83%	85%	76%	83%	82%	84%	84%
	[illegible]	[illegible]	[illegible]	[illegible]	[illegible]	[illegible]	[illegible]	[illegible]	[illegible]	[illegible]	[illegible]	[illegible]	[illegible]	[illegible]	[illegible]	[illegible]	[illegible]	[illegible]	[illegible]	[illegible]	[illegible]	[illegible]	[illegible]	[illegible]	[illegible]	[illegible]	[illegible]	[illegible]	[illegible]	[illegible]	[illegible]	[illegible]	[illegible]	[illegible]	[illegible]	[illegible]	[illegible]	[illegible]	[illegible]	[illegible]	[illegible]	[illegible]
Brown_trout_XP_029581515.1_	259	259	259	260	260	258	258	256	257	256	256	256	256	256	258	329	329	328	327	327	333	99%	90%	89%	93%	91%	91%	91%	91%	86%	81%	81%	82%	82%	82%	74%	78%	65%	75%	74%	78%	77%
	281	281	281	281	281	280	279	280	280	280	280	279	281	282	278	330	330	329	329	329	0	100%	93%	92%	95%	93%	93%	94%	93%	91%	86%	86%	87%	87%	87%	83%	85%	76%	83%	82%	84%	83%
	11	12	12	12	12	12	12	10	10	10	10	10	10	10	15	0	0	0	0	0	0	0%	1%	1%	0%	1%	1%	1%	2%	0%	2%	2%	2%	2%	2%	3%	3%	4%	4%	2%	4%	4%
Atlantic_salmon_XP_014045837.1	258	258	258	259	259	257	257	255	256	255	255	255	255	255	257	328	328	327	326	326	332	333	90%	89%	93%	91%	91%	91%	91%	86%	80%	81%	82%	82%	82%	75%	78%	65%	75%	74%	78%	77%
	281	281	281	281	281	280	279	280	280	280	280	279	281	282	278	330	330	329	329	329	333	0	93%	92%	95%	93%	93%	94%	93%	91%	86%	86%	87%	87%	87%	83%	85%	76%	83%	82%	84%	83%
	11	12	12	12	12	12	12	10	10	10	10	10	10	10	15	0	0	0	0	0	0	0	1%	1%	0%	1%	1%	1%	2%	0%	2%	2%	2%	2%	2%	3%	3%	4%	4%	2%	4%	4%
Brown_trout_XP_029562072.1_	254	254	254	255	255	255	255	251	250	250	250	249	251	251	253	303	303	302	303	301	301	300	329	94%	95%	93%	93%	93%	92%	84%	78%	78%	79%	79%	80%	73%	76%	64%	74%	73%	76%	75%
	278	278	278	278	278	277	276	278	277	279	279	277	280	281	273	312	312	311	313	311	310	310	0	95%	96%	94%	94%	94%	94%	90%	85%	85%	86%	86%	86%	83%	83%	76%	82%	81%	82%	81%
	15	16	16	16	16	16	16	14	14	14	14	14	14	14	19	4	4	4	4	4	4	4	0	2%	1%	2%	2%	2%	3%	1%	2%	2%	2%	2%	2%	3%	3%	5%	4%	2%	6%	5%
Atlantic_salmon_XP_013995955.1	255	255	255	256	256	257	257	254	252	252	252	251	252	252	254	304	304	303	304	302	302	301	320	338	94%	93%	94%	93%	93%	84%	77%	77%	78%	78%	78%	72%	74%	64%	72%	71%	76%	75%
	281	281	281	281	281	280	279	282	281	282	282	280	283	284	276	314	314	313	315	313	312	312	322	0	95%	95%	95%	95%	95%	89%	84%	84%	84%	84%	84%	82%	83%	76%	81%	80%	83%	82%
	6	7	7	7	7	7	7	7	7	7	7	7	7	7	10	5	5	5	5	5	5	5	9	0	1%	0%	0%	0%	0%	2%	3%	4%	4%	4%	4%	5%	5%	4%	5%	3%	3%	2%
Rainbow_trout_XP_021468992.1_	258	258	258	259	259	259	259	256	255	255	255	254	255	255	256	313	313	312	313	310	311	310	318	320	333	96%	97%	96%	96%	87%	80%	80%	81%	81%	82%	74%	76%	65%	75%	74%	78%	77%
	284	284	284	284	284	284	283	285	284	285	285	283	286	287	279	321	321	320	322	319	319	319	321	324	0	97%	97%	97%	97%	92%	87%	87%	88%	88%	88%	84%	84%	77%	83%	83%	84%	83%
	11	12	12	12	12	12	12	10	10	10	10	10	10	10	15	0	0	0	0	0	0	0	4	5	0	1%	1%	1%	2%	0%	2%	2%	2%	2%	2%	3%	3%	4%	4%	2%	4%	4%
Coho_salmon_XP_020322312.1_	261	261	261	262	262	261	261	259	259	259	259	258	259	259	259	312	312	311	311	309	310	309	316	318	327	339	99%	99%	98%	85%	79%	79%	80%	80%	81%	73%	76%	65%	73%	72%	79%	78%
	290	290	290	290	290	289	288	291	290	291	291	289	292	293	284	320	320	319	321	318	318	318	321	324	330	0	100%	99%	99%	90%	86%	85%	86%	86%	86%	83%	83%	76%	82%	81%	86%	84%
	9	10	10	10	10	10	10	8	8	8	8	8	8	8	13	6	6	6	6	6	6	6	10	3	6	0	0%	0%	0%	2%	4%	4%	4%	4%	4%	5%	5%	4%	6%	3%	4%	3%
Sockeye_salmon_XP_029497744.1_	263	263	263	264	264	263	263	260	260	260	260	259	260	260	261	313	313	312	313	310	311	310	318	320	329	337	339	99%	99%	85%	79%	79%	80%	80%	81%	73%	76%	65%	74%	73%	79%	78%
	290	290	290	290	290	289	288	291	290	291	291	289	292	293	284	320	320	319	321	318	318	318	321	324	330	339	0	99%	99%	90%	86%	85%	86%	86%	86%	83%	83%	76%	82%	81%	86%	84%
	9	10	10	10	10	10	10	8	8	8	8	8	8	8	13	6	6	6	6	6	6	6	10	3	6	0	0	0%	0%	2%	4%	4%	4%	4%	4%	5%	5%	4%	6%	3%	4%	3%
Chinook_salmon_XP_024292220.1_	262	262	262	263	263	262	262	260	260	260	260	259	260	260	260	313	313	312	311	310	311	310	316	318	327	336	337	339	98%	85%	79%	80%	80%	80%	81%	73%	76%	64%	74%	73%	79%	78%
	289	289	289	289	289	288	287	290	289	290	290	288	291	292	283	321	321	320	320	319	319	319	320	323	329	338	338	0	99%	91%	86%	86%	87%	87%	87%	83%	83%	76%	82%	82%	86%	85%
	9	10	10	10	10	10	10	8	8	8	8	8	8	8	13	6	6	6	6	6	6	6	10	3	6	0	0	0	0%	2%	4%	4%	4%	4%	4%	5%	5%	4%	6%	3%	4%	3%
Arctic_char_XP_023833474.1_	264	264	264	265	265	264	264	260	260	260	260	259	260	260	262	313	313	312	313	310	311	310	316	318	327	335	337	335	340	85%	79%	79%	80%	80%	80%	73%	75%	64%	73%	72%	79%	78%
	291	291	291	291	291	290	289	291	290	291	291	289	292	293	285	320	320	319	321	318	318	318	321	324	330	339	339	338	0	90%	85%	85%	86%	86%	86%	82%	83%	76%	81%	81%	86%	85%
	8	9	9	9	9	9	9	9	9	9	9	9	9	9	12	7	7	7	7	7	7	7	11	2	7	1	1	1	0	2%	4%	4%	4%	4%	4%	5%	5%	4%	6%	4%	4%	3%
Northern_pikeXP_010889688.1	254	254	254	255	255	255	255	251	253	249	249	248	249	250	250	291	291	290	289	289	290	290	283	285	291	290	291	292	290	333	79%	79%	79%	79%	80%	72%	75%	65%	74%	72%	75%	75%
	279	279	279	279	279	277	276	282	283	280	280	278	281	281	275	308	308	307	307	307	307	307	301	303	310	309	309	310	309	0	86%	86%	87%	87%	87%	81%	83%	77%	83%	80%	84%	82%
	13	14	14	14	14	14	14	12	12	12	12	12	12	12	17	2	2	2	2	2	2	2	6	7	2	8	8	8	9	0	2%	3%	3%	3%	3%	4%	4%	4%	4%	2%	5%	4%
Goldfish_XP_026063043.1_	260	260	260	261	260	259	259	255	257	256	256	253	256	254	257	271	271	272	270	270	270	269	260	261	268	269	270	271	270	264	325	98%	96%	95%	96%	75%	78%	66%	80%	76%	79%	81%
	287	287	287	287	286	286	286	284	285	283	283	281	284	283	280	290	290	291	290	290	289	289	284	286	291	292	292	293	292	290	0	98%	97%	97%	98%	85%	87%	79%	86%	84%	87%	88%
	15	16	16	16	16	16	16	14	14	14	14	14	14	14	13	8	8	8	8	8	8	8	8	13	8	14	14	14	15	10	0	0%	0%	0%	0%	3%	3%	5%	2%	2%	4%	2%
Common_carp_XP_018939072.1_	260	260	260	261	260	259	259	255	257	256	256	253	256	254	257	273	273	274	272	272	272	271	261	262	269	270	271	272	271	266	320	326	97%	96%	96%	75%	78%	67%	80%	76%	80%	81%
	287	287	287	287	286	286	287	284	285	283	283	281	284	283	280	290	290	291	290	290	289	289	284	286	291	292	292	293	292	290	322	0	98%	97%	99%	85%	86%	79%	86%	84%	88%	88%
	14	15	15	15	15	15	15	13	13	13	13	13	13	13	12	9	9	9	9	9	9	9	9	14	9	15	15	15	16	11	1	0	0%	0%	0%	3%	3%	5%	1%	1%	4%	2%
Goldfish_XP_026130800.1_	261	261	261	262	261	260	260	256	258	257	257	254	257	255	258	276	276	277	275	273	275	274	265	266	272	273	274	275	274	266	313	317	326	99%	96%	76%	77%	68%	81%	76%	80%	80%
	286	286	286	286	285	285	286	283	284	282	282	280	283	282	279	293	293	294	293	291	292	292	286	288	294	295	295	296	295	292	318	320	0	99%	99%	86%	86%	80%	88%	85%	88%	88%
	14	15	15	15	15	15	15	13	13	13	13	13	13	13	12	9	9	9	9	9	9	9	9	14	9	15	15	15	16	11	1	0	0	0%	0%	3%	3%	5%	1%	1%	4%	2%
Goldfish_XP_026106271.1_	260	260	260	261	260	259	259	255	257	256	256	253	256	255	258	276	276	277	275	273	275	274	265	266	272	273	274	275	274	266	312	316	325	326	96%	75%	77%	68%	81%	76%	80%	80%
	285	285	285	285	284	284	285	282	283	281	281	279	282	282	278	293	293	294	293	291	292	292	286	288	294	295	295	296	295	292	317	319	325	0	98%	86%	86%	79%	87%	85%	88%	88%
	14	15	15	15	15	15	15	13	13	13	13	13	13	13	12	9	9	9	9	9	9	9	9	14	9	15	15	15	16	11	1	0	0	0	0%	3%	3%	5%	1%	1%	4%	2%
Zebrafish_NP_775385.1_	261	261	261	262	261	260	260	256	258	257	257	254	257	255	258	277	277	278	276	274	276	275	266	267	275	276	277	278	276	269	313	316	316	315	326	76%	78%	68%	81%	76%	81%	82%
	286	286	286	286	285	285	286	283	284	282	282	280	283	282	279	293	293	294	293	291	292	292	286	288	294	295	295	296	295	292	321	323	323	322	0	86%	86%	80%	87%	85%	89%	88%
	14	15	15	15	15	15	15	13	13	13	13	13	13	13	12	9	9	9	9	9	9	9	9	14	9	15	15	15	16	11	1	0	0	0	0	3%	3%	5%	1%	1%	4%	2%
Stickleback_ENSGACT00000007360	263	263	263	262	261	261	261	254	255	256	256	253	255	256	259	252	252	252	251	250	251	252	246	247	249	252	251	252	251	243	252	252	253	252	253	326	87%	66%	70%	80%	76%	78%
	287	287	287	287	286	287	287	287	286	285	285	281	287	287	282	283	283	283	282	282	281	281	278	283	283	284	284	285	284	276	283	284	287	286	287	0	91%	78%	82%	89%	85%	85%
	14	15	15	15	15	15	15	13	13	13	13	13	13	13	18	13	13	13	13	13	13	13	13	18	13	19	19	19	20	15	13	12	12	12	12	0	1%	4%	5%	1%	5%	5%
Medaka_XP_004081243.1	271	271	271	271	271	269	269	265	266	267	267	264	266	268	270	261	261	261	260	259	260	261	253	253	256	259	258	259	258	253	258	258	257	257	259	286	321	67%	71%	80%	77%	80%
	292	292	292	292	291	291	291	289	290	287	287	284	289	289	288	286	286	286	285	285	284	284	278	281	282	283	283	284	283	278	287	287	286	286	285	298	0	78%	81%	87%	84%	84%
	19	20	20	20	20	20	20	18	18	18	18	18	18	18	23	12	12	12	12	12	12	12	12	17	12	18	18	18	19	14	12	13	13	13	13	5	0	5%	5%	3%	7%	6%
Human_NP_005903.2_	234	235	235	234	235	234	234	229	230	232	232	229	231	233	230	222	222	222	223	220	221	222	219	220	222	223	223	222	222	224	226	228	230	230	232	224	228	332	62%	66%	68%	67%
	275	276	276	276	277	275	276	272	272	273	273	270	273	273	267	262	262	262	263	261	261	261	259	261	263	263	263	262	263	263	268	269	271	270	271	263	265	0	74%	79%	80%	78%
	10	9	9	9	9	9	9	9	9	9	9	9	9	9	12	15	15	15	15	15	15	15	19	14	15	15	15	15	16	17	19	18	18	18	18	14	19	0	7%	3%	4%	3%
Cave_Fish_ENSAMXT00000027076	248	248	248	249	248	249	249	244	245	245	245	243	246	241	248	255	255	255	254	254	256	255	248	249	253	253	254	255	254	250	265	266	268	267	269	235	237	212	326	69%	73%	75%
	282	282	282	282	281	282	283	279	279	277	277	277	278	275	277	283	283	283	282	282	283	283	278	279	282	282	282	283	281	281	284	286	290	289	287	275	272	255	0	80%	83%	83%
	20	21	21	21	21	21	21	19	19	19	19	19	19	19	18	15	15	15	15	15	15	15	15	20	15	21	21	21	22	15	7	6	6	6	6	18	17	24	0	3%	4%	4%
Atlantic_cod_XP_030205462.1	260	260	260	261	262	261	262	257	256	258	258	255	258	259	258	251	251	251	250	249	249	250	245	245	249	249	250	251	250	244	254	253	255	254	254	267	268	224	233	332	75%	74%
	284	284	284	284	285	283	284	286	284	287	287	284	287	284	284	278	278	278	277	277	276	276	273	274	280	280	280	281	280	272	280	282	284	283	284	296	289	268	271	0	83%	82%
	8	9	9	9	9	9	9	7	7	7	7	7	7	7	12	7	7	7	7	7	7	7	7	12	7	13	13	13	14	9	7	6	6	6	6	6	11	12	12	0	4%	3%
Spotted_gar_ENSLOCP00000022262	276	276	276	277	278	275	275	270	272	272	272	269	270	268	268	269	269	269	268	267	268	267	261	262	268	274	273	274	273	260	268	270	272	271	274	261	262	233	248	257	334	82%
	300	300	300	299	301	299	299	297	300	298	298	295	299	297	293	290	290	290	289	289	289	289	282	285	289	297	297	298	298	289	294	297	298	297	300	291	288	273	283	283	0	90%
	6	5	5	5	5	5	5	7	7	7	7	7	7	7	2	17	17	17	17	17	17	17	21	12	17	15	15	15	14	19	15	14	14	14	14	20	25	14	16	14	0	1%
Asian_arowana__XP_018596452.1	277	277	277	277	277	275	275	270	269	271	271	268	272	273	272	266	266	267	265	264	265	264	257	258	263	269	268	269	268	259	272	272	271	270	275	265	272	231	254	252	278	335
	300	300	300	300	299	299	299	294	294	294	294	291	295	293	292	288	288	289	288	287	286	286	278	281	284	291	291	292	292	283	296	296	297	296	296	290	287	267	283	279	305	0
	5	6	6	6	6	6	6	6	6	6	6	6	6	6	3	14	14	14	14	14	14	14	18	9	14	12	12	12	11	16	10	9	9	9	9	17	22	13	15	11	5	0

Supplementary Table 4. Protein sequence identity matrix of human and Actinopterigii Pomc

	Human_POMC_NP_001030333.1_	Sockeye_salmon_POMC_XP_029534649.1_	Chinook_salmon_POMC_XP_024261009.1_	Rainbow_trout_POMCB_NP_001118191.1_	Arctic_char_POMCB_XP_023855328.1_	Atlantic_salmon_POMCB_NP_001122076.1_	Brown_trout_POMCB_XP_029602309.1_	Zebrafish_POMCB__NP_001076520_	Goldfish_POMC_XP_026141582.1_	Common_carp_Pomc2-like_XP_018920511.1_	Common_carp_Pomc1_XP_018947712.1_	Goldfish_POMC2-like_XP_026085507.1_	Zebrafish_Pomca_NP_852103.1_	Atlantic_salmon_POMCA1_NP_001185504.1_	Brown_trout_POMCA_XP_029586745.1_	Arctic_char_POMC_XP_023999323.1_	Sockeye_salmon_POMCA-like_XP_029504277.1	Coho_salmon_POMCA_XP_020328933.1_	Chinook_salmon_POMC_XP_024281382.1_	Sockeye_salmon_POMCA_XP_029503248.1_	Rainbow_trout_POMCA_NP_001118190.1_	Atlantic_salmon_POMCA2__NP_001185505.1_	Brown_trout_POMCA-like_XP_029568597.1_	Arctic_char_POMCA_XP_023999489.1_	Northern_pike_POMC_ENSELUP00000018832	Coho_salmon_Pomca-like_XP_031643588.1_	Asian_arowana_POMC1like_XP_029111572.1_	Atlantic_cod_Pomca_XP_030209683.1_	Coho_salmon_Pomc_XP_020311133.1_	Medaka_POMC_XP_004066504.1_	Stickleback_POMCA_ENSGACP00000012552_	Spotted_gar_POMC_ENSLOCP00000020612_	Asian_arowana_Pomca_XP_018586051.1_	Cave_fish_Pomc_ENSAMXP00000047531_	Northern_pike__ENSELUP00000063984	Medaka_POMCB_XP_004083656.1_	Atlantic_cod_Pomc-like_XP_030200870.1_	Goldfish_Pomc1-like_XP_026146178.1_	Goldfish_POMC1-like_XP_026104748.1	Common_carp_POMCB_ENSCCRP00000088364	Common_carp_Pomc1-like_XP_018928859.1_	Cave_fish_POMCB_ENSAMXP00000006682_	Stickleback_ENSGACP00000013340_
Human_POMC_NP_001030333.1_	267	31%	31%	30%	32%	31%	31%	28%	38%	39%	37%	37%	37%	37%	37%	38%	38%	37%	36%	37%	34%	37%	37%	37%	37%	38%	33%	33%	32%	33%	31%	37%	33%	38%	26%	23%	25%	26%	26%	21%	18%	24%	23%
	0	45%	45%	45%	47%	45%	46%	38%	49%	50%	47%	47%	50%	50%	50%	51%	51%	50%	51%	51%	47%	52%	51%	52%	50%	51%	44%	44%	46%	44%	43%	48%	45%	50%	38%	37%	35%	36%	36%	30%	25%	35%	34%
	0	20%	20%	21%	20%	20%	20%	24%	19%	19%	20%	20%	19%	18%	18%	17%	17%	12%	15%	15%	21%	15%	15%	15%	17%	17%	22%	27%	20%	23%	25%	22%	22%	20%	27%	21%	23%	28%	28%	37%	44%	28%	23%
Sockeye_salmon_POMC_XP_029534649.1_	86	226	97%	91%	94%	91%	92%	37%	39%	38%	38%	38%	37%	41%	40%	41%	41%	39%	40%	41%	37%	40%	40%	40%	41%	41%	35%	38%	97%	39%	33%	31%	36%	36%	48%	40%	33%	32%	32%	26%	25%	36%	38%
	126	0	98%	92%	95%	94%	94%	49%	54%	53%	55%	55%	53%	55%	54%	55%	55%	53%	55%	55%	50%	55%	55%	55%	54%	55%	54%	47%	98%	52%	49%	48%	51%	51%	64%	56%	45%	45%	45%	40%	35%	50%	49%
	55	0	0%	5%	0%	0%	0%	16%	14%	14%	13%	13%	14%	7%	7%	7%	6%	11%	9%	8%	16%	9%	9%	9%	7%	6%	11%	20%	0%	10%	17%	22%	17%	14%	16%	8%	9%	23%	23%	34%	37%	19%	11%
Chinook_salmon_POMC_XP_024261009.1_	87	220	226	90%	93%	90%	91%	37%	39%	39%	38%	38%	38%	41%	40%	41%	41%	39%	40%	41%	37%	40%	40%	40%	41%	41%	35%	38%	97%	38%	33%	31%	36%	36%	48%	39%	33%	32%	32%	26%	24%	35%	38%
	126	223	0	92%	95%	93%	94%	49%	53%	53%	54%	54%	53%	54%	53%	54%	54%	52%	54%	54%	50%	54%	54%	54%	54%	54%	53%	47%	98%	51%	49%	48%	51%	51%	64%	56%	46%	45%	45%	40%	35%	49%	50%
	55	0	0	5%	0%	0%	0%	16%	14%	14%	13%	13%	14%	7%	7%	7%	6%	11%	9%	8%	16%	9%	9%	9%	7%	6%	11%	20%	0%	10%	17%	22%	17%	14%	16%	8%	9%	23%	23%	34%	37%	19%	11%
Rainbow_trout_POMCB_NP_001118191.1_	88	219	218	240	90%	87%	88%	35%	39%	38%	38%	38%	37%	39%	38%	39%	39%	38%	39%	40%	36%	38%	39%	39%	39%	39%	33%	36%	91%	37%	32%	32%	35%	36%	45%	38%	31%	32%	32%	27%	23%	36%	35%
	129	222	221	0	90%	89%	90%	46%	54%	53%	54%	54%	53%	53%	52%	52%	52%	51%	53%	53%	49%	53%	53%	53%	52%	52%	52%	45%	92%	50%	47%	48%	50%	51%	60%	55%	43%	47%	47%	41%	32%	52%	47%
	61	14	14	0	5%	6%	6%	20%	14%	14%	13%	13%	14%	12%	13%	12%	11%	16%	14%	13%	20%	14%	14%	14%	12%	11%	16%	24%	6%	15%	21%	21%	21%	16%	21%	13%	14%	18%	18%	30%	40%	14%	15%
Arctic_char_POMCB_XP_023855328.1_	90	213	212	218	226	95%	95%	38%	41%	41%	40%	40%	39%	42%	41%	41%	42%	40%	42%	42%	38%	41%	42%	42%	42%	42%	35%	39%	95%	40%	34%	33%	38%	38%	49%	41%	34%	33%	33%	27%	24%	37%	38%
	131	216	215	218	0	96%	96%	51%	57%	56%	57%	57%	56%	56%	56%	56%	56%	54%	56%	56%	52%	56%	56%	56%	55%	56%	57%	49%	96%	54%	52%	50%	53%	54%	65%	58%	47%	46%	46%	41%	34%	51%	50%
	55	0	0	14	0	0%	0%	16%	14%	14%	13%	13%	14%	7%	7%	7%	6%	11%	9%	8%	16%	9%	9%	9%	7%	6%	11%	20%	0%	10%	17%	22%	17%	14%	16%	8%	9%	23%	23%	34%	37%	19%	11%
Atlantic_salmon_POMCB_NP_001122076.1_	86	206	205	211	215	225	96%	36%	40%	39%	39%	39%	38%	41%	40%	41%	40%	40%	41%	42%	37%	40%	41%	41%	41%	40%	34%	38%	92%	38%	33%	31%	36%	37%	49%	40%	33%	31%	31%	26%	23%	35%	38%
	125	213	212	215	219	0	97%	48%	54%	53%	55%	55%	53%	55%	54%	55%	54%	53%	55%	55%	50%	55%	55%	55%	54%	54%	55%	47%	94%	52%	50%	47%	51%	52%	64%	58%	47%	44%	44%	39%	33%	50%	49%
	56	1	1	15	1	0	0%	16%	14%	14%	13%	13%	14%	7%	8%	7%	7%	12%	9%	8%	16%	9%	9%	9%	7%	7%	12%	20%	0%	10%	17%	22%	17%	14%	16%	8%	9%	23%	23%	35%	37%	19%	11%
Brown_trout_POMCB_XP_029602309.1_	87	208	207	213	215	217	225	37%	40%	39%	39%	39%	38%	41%	40%	41%	41%	40%	41%	42%	37%	40%	41%	41%	41%	41%	35%	38%	93%	38%	34%	32%	36%	37%	48%	40%	32%	32%	32%	26%	24%	36%	37%
	128	214	213	216	218	220	0	49%	54%	54%	55%	55%	53%	55%	54%	54%	54%	53%	55%	55%	50%	55%	55%	55%	55%	54%	54%	47%	95%	52%	50%	48%	51%	51%	64%	58%	46%	45%	45%	40%	34%	50%	49%
	56	1	1	15	1	0	0	16%	14%	14%	13%	13%	14%	7%	8%	7%	7%	12%	9%	8%	16%	9%	9%	9%	7%	7%	12%	20%	0%	10%	17%	22%	17%	14%	16%	8%	9%	23%	23%	35%	37%	19%	11%
Zebrafish_POMCB__NP_001076520_	77	90	90	89	93	88	89	215	36%	36%	35%	35%	34%	36%	35%	35%	35%	35%	36%	37%	33%	36%	36%	37%	34%	35%	32%	31%	38%	30%	32%	30%	33%	34%	27%	23%	27%	68%	68%	55%	51%	36%	26%
	107	119	119	119	123	116	119	0	50%	49%	49%	49%	48%	47%	46%	46%	47%	46%	47%	48%	44%	47%	48%	48%	45%	47%	48%	43%	50%	43%	45%	46%	46%	48%	39%	36%	36%	79%	78%	65%	62%	47%	36%
	68	39	39	53	39	40	40	0	15%	15%	16%	16%	14%	16%	17%	16%	16%	16%	14%	13%	21%	14%	14%	14%	17%	16%	12%	20%	16%	22%	18%	23%	18%	16%	24%	20%	22%	11%	11%	25%	28%	23%	22%
Goldfish_POMC_XP_026141582.1_	104	95	96	99	100	97	98	86	222	93%	88%	88%	82%	56%	56%	56%	56%	54%	57%	56%	52%	56%	57%	57%	55%	56%	45%	47%	40%	47%	50%	51%	49%	64%	27%	32%	28%	34%	34%	28%	24%	34%	28%
	135	132	130	136	138	131	132	119	0	95%	91%	91%	90%	68%	68%	67%	68%	68%	70%	69%	65%	70%	71%	71%	67%	68%	56%	60%	55%	60%	66%	63%	60%	73%	41%	50%	42%	47%	47%	39%	32%	50%	44%
	53	34	34	36	34	35	35	37	0	0%	1%	1%	0%	13%	13%	14%	13%	12%	10%	10%	17%	10%	10%	10%	12%	13%	11%	14%	14%	19%	10%	17%	13%	7%	25%	15%	21%	18%	18%	31%	39%	15%	19%
Common_carp_Pomc2-like_XP_018920511.1_	106	93	94	97	99	95	96	86	208	222	89%	89%	80%	54%	54%	54%	54%	52%	55%	54%	50%	54%	55%	55%	55%	54%	44%	47%	40%	47%	50%	49%	49%	62%	27%	31%	28%	33%	33%	27%	23%	34%	27%
	137	130	128	134	137	130	131	118	213	0	92%	92%	89%	67%	66%	65%	66%	67%	69%	69%	64%	69%	70%	70%	66%	66%	55%	60%	54%	58%	65%	62%	61%	73%	41%	49%	41%	47%	47%	39%	31%	50%	42%
	53	34	34	36	34	35	35	37	0	0	1%	1%	0%	13%	13%	14%	13%	12%	10%	10%	17%	10%	10%	10%	12%	13%	11%	14%	14%	19%	10%	17%	13%	7%	25%	15%	21%	18%	18%	31%	39%	15%	19%
Common_carp_Pomc1_XP_018947712.1_	101	91	92	95	97	93	94	85	196	198	219	100%	78%	53%	52%	52%	52%	51%	53%	53%	49%	53%	53%	53%	54%	52%	43%	47%	39%	46%	48%	48%	47%	60%	27%	30%	27%	32%	32%	27%	23%	33%	28%
	129	131	129	135	138	131	132	118	204	206	0	100%	86%	65%	64%	63%	63%	63%	65%	64%	60%	66%	66%	66%	63%	63%	55%	60%	55%	59%	63%	61%	60%	71%	42%	50%	41%	46%	46%	38%	31%	50%	44%
	56	31	31	33	31	32	32	40	3	3	0	0%	2%	15%	14%	15%	15%	13%	11%	12%	18%	11%	11%	11%	13%	15%	10%	14%	13%	18%	11%	19%	14%	8%	25%	17%	20%	20%	20%	32%	39%	14%	20%
Goldfish_POMC2-like_XP_026085507.1_	101	91	92	95	97	93	94	85	196	198	219	219	78%	53%	52%	52%	52%	51%	53%	53%	49%	53%	53%	53%	54%	52%	43%	47%	39%	46%	48%	48%	47%	60%	27%	30%	27%	32%	32%	27%	23%	33%	28%
	129	131	129	135	138	131	132	118	204	206	219	0	86%	65%	64%	63%	63%	63%	65%	64%	60%	66%	66%	66%	63%	63%	55%	60%	55%	59%	63%	61%	60%	71%	42%	50%	41%	46%	46%	38%	31%	50%	44%
	56	31	31	33	31	32	32	40	3	3	0	0	2%	15%	14%	15%	15%	13%	11%	12%	18%	11%	11%	11%	13%	15%	10%	14%	13%	18%	11%	19%	14%	8%	25%	17%	20%	20%	20%	32%	39%	14%	20%
Zebrafish_Pomca_NP_852103.1_	102	90	92	94	94	92	93	82	185	179	176	176	222	55%	54%	54%	54%	52%	55%	54%	50%	55%	55%	55%	54%	54%	44%	47%	38%	46%	49%	50%	47%	61%	27%	31%	27%	33%	33%	27%	24%	34%	28%
	138	129	128	133	135	129	130	114	202	199	193	193	0	65%	64%	63%	64%	63%	66%	65%	61%	66%	66%	66%	62%	64%	54%	59%	54%	57%	63%	61%	59%	71%	41%	50%	40%	44%	44%	38%	30%	49%	44%
	53	34	34	36	34	35	35	35	2	2	5	5	0	13%	13%	14%	13%	12%	10%	10%	17%	10%	10%	10%	12%	13%	11%	14%	14%	19%	10%	17%	13%	7%	25%	15%	21%	18%	18%	30%	39%	16%	19%
Atlantic_salmon_POMCA1_NP_001185504.1_	104	98	98	100	100	98	98	89	139	134	131	131	135	232	98%	92%	90%	78%	80%	81%	74%	83%	82%	80%	77%	91%	42%	50%	42%	56%	52%	51%	47%	54%	28%	35%	26%	33%	33%	27%	23%	30%	32%
	138	132	130	134	135	131	131	116	168	164	159	159	160	0	98%	93%	91%	82%	84%	84%	78%	87%	86%	84%	82%	91%	55%	59%	56%	64%	63%	62%	57%	66%	43%	52%	41%	44%	44%	37%	30%	41%	47%
	51	18	18	32	18	19	19	41	34	34	37	37	34	0	0%	1%	2%	8%	5%	5%	13%	5%	5%	5%	2%	2%	14%	21%	7%	11%	16%	20%	15%	10%	22%	8%	13%	23%	23%	34%	39%	23%	12%
Brown_trout_POMCA_XP_029586745.1_	104	96	96	98	98	96	96	88	137	132	129	129	132	229	233	91%	89%	78%	81%	81%	74%	83%	83%	80%	76%	90%	42%	50%	41%	55%	52%	51%	47%	53%	28%	35%	26%	32%	32%	26%	22%	29%	32%
	138	131	129	133	134	130	130	115	166	162	157	157	157	229	0	92%	91%	82%	84%	84%	78%	87%	86%	84%	81%	91%	55%	59%	55%	63%	62%	62%	57%	66%	42%	51%	40%	43%	43%	36%	30%	40%	46%
	50	19	19	33	19	20	20	42	33	33	36	36	33	1	0	2%	2%	8%	5%	6%	13%	5%	5%	5%	2%	2%	14%	20%	8%	12%	15%	20%	14%	10%	22%	8%	13%	24%	24%	34%	39%	23%	12%
Arctic_char_POMC_XP_023999323.1_	107	98	98	100	100	98	98	87	138	133	130	130	133	218	217	234	94%	80%	82%	84%	75%	84%	84%	83%	77%	94%	43%	50%	42%	54%	51%	51%	47%	54%	28%	34%	26%	32%	32%	26%	22%	30%	32%
	141	132	130	134	135	132	131	115	165	162	156	156	157	220	219	0	95%	83%	86%	87%	80%	88%	88%	87%	82%	95%	55%	58%	55%	63%	61%	61%	58%	66%	42%	51%	42%	43%	43%	36%	30%	41%	47%
	49	18	18	32	18	19	19	41	36	36	39	39	36	4	5	0	0%	7%	5%	4%	12%	5%	5%	5%	2%	0%	13%	21%	7%	11%	16%	20%	15%	11%	22%	9%	11%	23%	23%	34%	39%	23%	13%
Sockeye_salmon_POMCA-like_XP_029504277.1_	106	98	98	100	100	97	98	87	139	134	129	129	134	213	212	221	232	80%	82%	85%	75%	84%	85%	83%	75%	99%	43%	50%	42%	55%	52%	51%	47%	54%	28%	34%	25%	32%	32%	26%	21%	30%	32%
	140	131	129	133	134	130	130	115	166	163	155	155	158	216	215	223	0	84%	86%	88%	80%	88%	89%	86%	80%	100%	56%	59%	55%	64%	62%	61%	58%	67%	42%	50%	41%	43%	43%	36%	29%	42%	47%
	47	16	16	30	16	17	17	39	34	34	37	37	34	6	7	2	0	6%	4%	3%	12%	4%	4%	4%	2%	0%	13%	21%	7%	10%	16%	19%	15%	12%	21%	10%	12%	23%	23%	33%	39%	22%	12%
Coho_salmon_POMCA_XP_020328933.1_	100	98	98	100	101	99	99	89	135	130	126	126	130	194	194	197	197	239	94%	92%	86%	91%	92%	89%	71%	81%	42%	51%	40%	51%	51%	51%	48%	52%	28%	32%	26%	32%	32%	26%	22%	30%	30%
	137	132	130	134	134	131	131	115	168	166	156	156	156	204	203	206	206	0	94%	93%	87%	92%	94%	92%	78%	84%	54%	59%	54%	60%	61%	61%	59%	64%	42%	48%	39%	42%	42%	36%	30%	40%	45%
	34	29	29	43	29	30	30	42	31	31	34	34	31	21	20	19	17	0	2%	3%	10%	2%	2%	2%	7%	6%	12%	18%	12%	15%	15%	18%	13%	13%	23%	13%	17%	23%	23%	34%	39%	23%	16%
Chinook_salmon_POMC_XP_024281382.1_	99	98	98	100	101	99	99	89	137	132	128	128	132	193	194	196	196	225	232	95%	89%	94%	95%	92%	73%	83%	43%	52%	41%	52%	53%	52%	50%	54%	29%	33%	27%	33%	33%	27%	23%	31%	31%
	139	133	131	135	136	133	132	115	169	166	156	156	159	203	203	206	206	227	0	96%	90%	96%	97%	95%	80%	86%	56%	61%	56%	61%	64%	62%	60%	66%	43%	49%	40%	44%	44%	37%	30%	41%	47%
	41	22	22	36	22	23	23	35	24	24	27	27	24	14	13	12	10	7	0	0%	8%	0%	0%	0%	4%	4%	10%	17%	9%	13%	13%	16%	12%	10%	21%	11%	14%	21%	21%	32%	37%	21%	13%
Sockeye_salmon_POMCA_XP_029503248.1_	100	99	99	101	102	100	100	90	136	131	127	127	131	195	195	200	200	222	222	230	88%	94%	95%	92%	72%	85%	43%	52%	42%	53%	52%	51%	49%	54%	29%	33%	26%	33%	33%	27%	23%	31%	31%
	139	132	130	134	135	132	131	115	167	165	155	155	156	202	202	207	207	224	224	0	89%	95%	96%	94%	78%	88%	56%	61%	56%	62%	64%	62%	60%	66%	43%	49%	40%	43%	43%	37%	30%	41%	46%
	43	20	20	34	20	21	21	33	26	26	29	29	26	14	15	10	8	9	2	0	9%	0%	0%	0%	5%	3%	10%	18%	8%	12%	13%	17%	12%	11%	20%	11%	14%	21%	21%	32%	37%	20%	13%
Rainbow_trout_POMCA_NP_001118190.1_	101	97	97	99	100	98	98	89	137	132	128	128	132	193	194	196	196	224	226	223	253	88%	88%	85%	67%	76%	40%	48%	38%	47%	49%	48%	47%	50%	27%	30%	25%	31%	31%	26%	21%	28%	34%
	139	133	131	135	136	133	132	116	170	168	158	158	159	205	205	208	208	228	229	227	0	89%	90%	88%	73%	80%	52%	56%	51%	56%	59%	58%	57%	61%	40%	46%	37%	40%	40%	36%	28%	38%	51%
	62	43	43	57	43	44	44	56	45	45	48	48	45	35	34	33	31	28	21	23	0	8%	8%	8%	12%	12%	17%	24%	16%	20%	20%	22%	16%	17%	27%	18%	21%	27%	27%	35%	42%	27%	5%
Atlantic_salmon_POMCA2__NP_001185505.1_	102	97	97	99	100	98	98	88	136	131	129	129	132	199	200	203	201	219	220	220	223	232	98%	95%	74%	85%	43%	53%	41%	52%	52%	52%	50%	53%	28%	34%	27%	32%	32%	27%	23%	31%	31%
	141	133	131	135	136	133	132	115	168	166	158	158	158	208	208	212	210	222	223	222	226	0	98%	96%	80%	88%	55%	61%	56%	61%	64%	63%	60%	66%	43%	50%	41%	43%	43%	37%	30%	41%	47%
	41	22	22	36	22	23	23	35	24	24	27	27	24	14	13	12	10	7	0	2	21	0	0%	0%	4%	4%	10%	17%	9%	13%	13%	16%	12%	10%	21%	11%	14%	21%	21%	32%	37%	21%	13%
Brown_trout_POMCA-like_XP_029568597.1_	101	98	98	100	101	99	99	89	138	133	129	129	133	198	199	202	202	221	222	222	225	229	232	96%	74%	85%	44%	53%	41%	52%	53%	53%	51%	54%	29%	33%	27%	33%	33%	27%	23%	31%	31%
	140	133	131	135	136	133	132	117	170	168	158	158	159	207	207	211	211	225	226	225	229	229	0	97%	81%	89%	56%	61%	56%	62%	64%	63%	61%	67%	43%	50%	41%	44%	44%	37%	30%	41%	46%
	41	22	22	36	22	23	23	35	24	24	27	27	24	14	13	12	10	7	0	2	21	0	0	0%	4%	4%	10%	17%	9%	13%	13%	16%	12%	10%	21%	11%	14%	21%	21%	32%	37%	21%	13%
Arctic_char_POMCA_XP_023999489.1_	101	98	98	100	101	99	99	90	138	133	129	129	133	192	193	200	197	214	215	214	217	221	224	232	74%	83%	44%	52%	41%	51%	52%	52%	52%	53%	29%	33%	27%	33%	33%	27%	23%	31%	31%
	141	133	131	135	136	133	132	117	170	168	158	158	159	202	202	210	206	221	222	220	224	224	227	0	80%	86%	57%	61%	56%	60%	63%	63%	61%	67%	43%	49%	41%	44%	44%	37%	30%	42%	46%
	41	22	22	36	22	23	23	35	24	24	27	27	24	14	13	12	10	7	0	2	21	0	0	0	4%	4%	10%	17%	9%	13%	13%	16%	12%	10%	21%	11%	14%	21%	21%	32%	37%	21%	13%
Northern_pike_POMC_ENSELUP00000018832_	104	99	100	101	102	100	100	86	136	136	132	132	132	183	181	184	179	175	176	174	175	177	179	177	235	75%	41%	48%	42%	54%	51%	51%	48%	52%	28%	32%	25%	31%	31%	26%	20%	30%	31%
	138	131	130	133	133	130	133	113	164	163	156	156	153	196	194	195	191	193	192	188	192	192	194	193	0	80%	53%	58%	55%	61%	61%	61%	57%	65%	41%	50%	38%	41%	41%	35%	28%	41%	45%
	48	17	17	31	17	18	18	42	31	31	34	34	31	7	6	5	7	18	11	13	32	11	11	11	0	2%	11%	20%	7%	11%	15%	19%	14%	10%	22%	7%	12%	24%	24%	34%	39%	24%	11%
Coho_salmon_Pomca-like_XP_031643588.1_	106	98	98	100	100	97	98	87	139	134	129	129	134	214	213	220	230	198	197	201	197	202	203	197	179	232	43%	50%	42%	55%	52%	51%	48%	54%	28%	34%	25%	32%	32%	26%	21%	30%	32%
	140	131	129	133	134	130	130	115	166	163	155	155	158	216	215	223	232	206	206	207	208	210	211	206	191	0	56%	59%	55%	64%	62%	61%	58%	66%	42%	50%	41%	43%	43%	36%	29%	41%	47%
	47	16	16	30	16	17	17	39	34	34	37	37	34	6	7	2	0	17	10	8	31	10	10	10	7	0	13%	21%	7%	10%	16%	19%	15%	12%	21%	10%	12%	23%	23%	33%	39%	22%	12%
Asian_arowana_POMC1like_XP_029111572.1_	92	85	84	84	86	83	84	77	108	106	101	101	106	104	104	106	107	104	105	105	107	105	106	107	101	107	224	36%	35%	40%	38%	38%	43%	44%	26%	28%	26%	29%	29%	24%	22%	31%	28%
	122	131	129	132	137	132	131	113	135	131	131	131	128	136	136	136	137	135	136	135	136	134	135	137	131	137	0	49%	55%	50%	52%	50%	52%	54%	42%	46%	40%	44%	44%	35%	32%	44%	41%
	61	28	28	42	28	29	29	29	28	28	25	25	28	36	35	34	32	31	24	26	45	24	24	24	29	32	0	19%	12%	16%	14%	21%	15%	12%	24%	16%	18%	20%	20%	32%	34%	21%	19%
Atlantic_cod_Pomca_XP_030209683.1_	93	93	93	94	96	93	93	75	111	111	110	110	112	126	125	126	126	127	128	128	128	130	130	129	122	126	89	213	38%	46%	48%	37%	41%	44%	28%	30%	25%	27%	27%	23%	22%	28%	31%
	123	117	117	118	121	117	116	103	141	141	141	141	140	148	147	148	148	148	150	149	150	151	151	150	145	148	120	0	48%	54%	61%	49%	54%	56%	40%	43%	35%	39%	39%	34%	29%	41%	41%
	76	49	49	63	49	50	50	48	33	33	34	34	35	53	52	55	53	46	43	45	64	43	43	43	52	53	47	0	20%	25%	15%	28%	18%	19%	28%	21%	25%	27%	27%	37%	42%	21%	25%
Coho_salmon_Pomc_XP_020311133.1_	89	222	221	221	217	210	212	92	99	97	95	95	93	101	99	101	101	101	101	102	100	100	101	101	102	101	85	95	227	39%	34%	32%	37%	38%	48%	40%	33%	32%	32%	27%	25%	36%	38%
	128	224	223	222	218	215	216	121	134	132	133	133	131	134	133	134	133	134	135	134	135	135	135	135	133	133	133	119	0	53%	50%	48%	52%	52%	64%	57%	45%	46%	46%	41%	35%	50%	49%
	56	1	1	15	1	2	2	40	35	35	32	32	35	19	20	19	17	30	23	21	44	23	23	23	18	17	29	50	0	10%	16%	22%	17%	14%	17%	8%	10%	23%	23%	35%	37%	19%	11%
Medaka_POMC_XP_004066504.1_	92	91	90	91	93	90	90	74	115	113	111	111	111	133	131	129	131	127	125	126	124	124	125	122	128	131	96	112	92	212	54%	41%	42%	44%	27%	31%	28%	26%	26%	21%	19%	29%	29%
	121	122	120	124	126	121	122	104	144	141	140	140	138	153	150	150	151	148	146	147	147	147	148	145	145	151	121	132	124	0	67%	53%	53%	56%	41%	47%	41%	37%	37%	31%	28%	39%	41%
	65	24	24	38	24	25	25	53	46	46	43	43	46	28	29	26	24	39	32	30	53	32	32	32	27	24	40	61	25	0	13%	26%	19%	14%	25%	17%	15%	28%	28%	39%	43%	26%	19%
Stickleback_POMCA_ENSGACP00000012552_	86	79	79	81	83	80	81	76	115	115	109	109	112	126	125	124	126	124	127	125	126	125	126	123	123	125	90	112	82	123	209	40%	43%	45%	26%	27%	27%	27%	27%	23%	20%	29%	29%
	118	118	117	120	124	119	119	107	151	149	145	145	145	153	151	149	151	150	152	152	153	152	153	151	148	151	123	141	121	153	0	53%	58%	60%	38%	45%	40%	39%	39%	33%	28%	44%	41%
	68	41	41	55	41	42	42	44	23	23	26	26	23	39	38	41	39	38	31	33	52	31	31	31	38	39	35	36	40	31	0	24%	13%	13%	28%	18%	22%	25%	25%	37%	41%	21%	21%
Spotted_gar_POMC_ENSLOCP00000020612_	113	87	86	92	92	88	90	82	138	133	131	131	134	144	143	142	141	143	143	141	143	144	145	144	142	141	106	104	90	114	110	266	45%	49%	25%	28%	24%	27%	27%	23%	19%	28%	26%
	147	134	133	138	139	132	133	127	171	167	165	165	164	174	174	171	170	171	171	169	171	172	172	173	170	170	139	139	136	147	144	0	54%	59%	38%	44%	37%	43%	43%	37%	30%	40%	39%
	69	62	62	62	62	63	63	65	48	48	51	51	48	58	57	56	54	53	46	48	67	46	46	46	53	54	58	79	63	72	67	0	23%	18%	29%	23%	27%	25%	25%	35%	44%	26%	25%
Asian_arowana_Pomca_XP_018586051.1_	94	92	91	94	96	92	92	83	121	120	117	117	117	119	119	120	121	123	125	123	125	125	127	129	122	122	108	103	95	104	103	128	234	50%	27%	31%	25%	30%	30%	26%	19%	30%	27%
	130	131	130	133	136	131	130	116	148	149	147	147	145	146	146	148	149	150	151	149	152	150	152	152	146	149	131	133	134	133	138	155	0	60%	41%	46%	39%	42%	42%	37%	30%	42%	42%
	65	44	44	58	44	45	45	47	32	32	35	35	32	38	37	40	40	33	30	32	43	30	30	30	37	40	38	45	45	48	31	68	0	11%	29%	18%	23%	25%	25%	33%	42%	25%	18%
Cave_fish_Pomc_ENSAMXP00000047531_	104	88	89	92	93	91	91	81	148	144	138	138	142	130	129	131	132	129	131	131	130	129	131	129	127	132	106	107	92	104	105	134	121	221	26%	33%	28%	31%	31%	27%	21%	33%	25%
	136	125	124	129	131	126	125	114	170	169	164	164	164	160	159	162	162	159	160	160	161	159	161	161	159	161	129	136	127	133	139	161	146	0	40%	47%	40%	44%	44%	38%	30%	46%	41%
	56	35	35	41	35	36	36	38	17	17	20	20	17	25	24	29	29	32	25	27	46	25	25	25	26	29	29	46	36	35	30	51	29	0	27%	15%	21%	20%	20%	32%	40%	20%	18%
Northern_pike__ENSELUP00000063984_	72	112	112	113	115	114	112	65	68	66	67	67	67	70	69	70	70	71	71	71	72	70	71	71	71	70	63	70	114	65	63	70	71	65	201	31%	26%	24%	24%	19%	23%	23%	28%
	105	150	151	150	152	150	150	94	102	101	101	101	101	105	105	104	104	105	105	104	106	106	106	106	102	104	103	97	151	99	92	106	105	99	0	46%	37%	37%	37%	32%	35%	38%	39%
	76	39	39	53	39	38	38	58	63	63	60	60	63	55	56	55	53	58	51	49	72	51	51	51	56	53	59	70	40	59	68	81	75	66	0	23%	22%	31%	31%	41%	33%	29%	25%
Medaka_POMCB_XP_004083656.1_	66	96	93	97	98	96	95	58	79	77	75	75	76	85	85	84	84	82	81	81	81	83	82	82	79	84	70	75	97	76	67	79	80	81	77	227	28%	21%	21%	19%	19%	26%	41%
	105	134	134	138	139	137	138	91	124	121	123	123	122	125	123	124	123	122	121	120	122	123	122	121	121	123	114	107	137	113	110	125	119	116	113	0	39%	34%	34%	30%	28%	38%	53%
	60	19	19	33	19	20	20	50	39	39	42	42	39	21	20	23	25	34	27	29	48	27	27	27	18	25	41	54	20	41	44	67	47	38	58	0	15%	27%	27%	37%	41%	25%	13%
Atlantic_cod_Pomc-like_XP_030200870.1_	70	79	79	79	81	78	77	69	72	71	69	69	68	66	65	64	63	67	67	66	68	67	67	67	63	63	66	64	79	67	67	69	65	70	64	70	225	24%	24%	18%	19%	24%	27%
	**99**	109	110	110	112	111	109	90	106	105	104	104	101	101	100	103	101	100	101	100	101	102	102	102	94	101	99	89	109	99	99	106	103	101	91	97	0	34%	34%	27%	28%	37%	37%
	**64**	23	23	37	23	22	22	56	55	55	52	52	55	33	34	29	31	44	37	35	58	37	37	37	30	31	45	64	24	37	56	79	61	54	54	38	0	29%	29%	39%	42%	24%	19%
Goldfish_Pomc1-like_XP_026146178.1_	77	85	86	87	89	83	85	165	87	86	84	84	85	89	88	87	87	89	89	88	89	87	88	89	85	87	77	72	87	69	70	81	82	80	64	59	67	240	99%	69%	55%	38%	27%
	107	121	121	125	124	118	120	191	121	120	118	118	114	118	117	116	116	116	117	115	117	116	117	117	111	116	114	103	122	99	103	126	116	115	98	92	93	0	99%	74%	58%	50%	35%
	83	62	62	48	62	63	63	27	48	48	51	51	46	64	65	64	62	65	58	56	79	58	58	58	65	62	52	71	63	76	67	74	70	53	83	73	79	0	0%	16%	35%	15%	28%
Goldfish_POMC1-like_XP_026104748.1	77	85	86	87	89	83	85	164	87	86	84	84	85	89	88	87	87	89	89	88	89	87	88	89	85	87	77	72	87	69	70	81	82	80	64	59	67	239	240	69%	55%	38%	27%
	107	121	121	125	124	118	120	190	121	120	118	118	114	118	117	116	116	116	117	115	117	116	117	117	111	116	114	103	122	99	103	126	116	115	98	92	93	239	0	74%	58%	50%	35%
	83	62	62	48	62	63	63	27	48	48	51	51	46	64	65	64	62	65	58	56	79	58	58	58	65	62	52	71	63	76	67	74	70	53	83	73	79	0	0	16%	35%	15%	28%
Common_carp_POMCB_ENSCCRP00000088364_	75	83	82	85	86	81	82	158	85	84	82	82	82	85	84	84	84	85	85	85	87	84	85	86	83	84	73	71	86	67	70	79	82	82	61	61	59	199	198	286	42%	32%	22%
	105	126	125	130	128	122	124	187	120	119	117	117	114	116	115	114	115	115	116	115	118	115	116	116	112	115	109	106	128	99	103	125	117	117	100	96	86	214	213	0	47%	43%	30%
	129	108	108	94	108	109	109	73	94	94	97	97	92	108	109	108	106	109	102	100	115	102	102	102	109	106	98	117	109	122	113	118	106	97	129	119	125	46	46	0	45%	28%	35%
Common_carp_Pomc1-like_XP_018928859.1_	49	59	58	58	58	54	57	112	58	56	55	55	57	56	55	54	53	56	56	55	56	55	55	56	51	53	52	52	59	46	47	52	49	51	50	48	47	134	134	123	156	25%	19%
	69	83	83	82	82	79	81	135	77	75	74	74	71	75	74	73	72	75	74	72	74	74	74	74	69	72	75	70	83	66	65	82	75	71	77	68	70	141	141	136	0	33%	26%
	121	88	88	102	88	87	87	61	94	94	91	91	92	96	97	96	94	97	90	88	111	90	90	90	97	94	78	99	89	102	97	122	104	95	71	99	103	86	86	132	0	43%	44%
Cave_fish_POMCB_ENSAMXP00000006682_	73	93	90	94	95	91	93	93	86	86	83	83	86	81	79	80	81	83	83	83	82	82	83	83	83	81	81	72	94	75	74	83	82	85	61	71	65	99	99	98	64	236	28%
	103	128	127	134	132	128	130	122	125	125	124	124	124	110	108	110	111	108	110	109	110	109	110	111	110	110	115	103	130	102	110	117	115	119	99	103	99	129	129	132	83	0	36%
	83	50	50	36	50	51	51	61	38	38	35	35	40	62	63	62	60	63	56	54	77	56	56	56	65	60	54	53	51	68	53	76	68	53	75	69	65	40	40	86	110	0	27%
Stickleback_ENSGACP00000013340_	70	98	98	96	98	97	96	70	74	73	74	74	76	84	84	85	84	83	83	82	91	83	83	83	82	84	75	83	98	77	77	78	75	68	75	106	73	80	80	73	52	80	254
	103	126	127	126	128	125	125	97	116	113	118	118	116	123	121	124	123	121	123	122	135	123	122	122	119	123	111	111	127	107	108	119	113	110	102	139	100	102	102	101	70	105	0
	71	28	28	42	28	29	29	59	50	50	53	53	50	32	33	34	32	43	36	36	15	36	36	36	31	32	52	67	29	50	57	76	50	49	67	35	51	82	82	118	116	78	0
